# Morphoagronomic characterization and whole-genome resequencing of eight highly diverse wild and weedy *S. pimpinellifolium* and *S. lycopersicum* var. *cerasiforme* accessions used for the first interspecific tomato MAGIC population

**DOI:** 10.1038/s41438-020-00395-w

**Published:** 2020-11-01

**Authors:** Pietro Gramazio, Leandro Pereira-Dias, Santiago Vilanova, Jaime Prohens, Salvador Soler, Javier Esteras, Alfonso Garmendia, María José Díez

**Affiliations:** 1 Faculty of Life and Environmental Sciences, University of Tsukuba, 1-1-1 Tennodai, 305-8572 Tsukuba, Japan; 2 Instituto de Conservación y Mejora de la Agrodiversidad Valenciana, Universitat Politècnica de València, Camino de Vera 14, 46022 Valencia, Spain; 3 Departamento de Ecosistemas Agroforestales, Universitat Politècnica de València, Camino de Vera 14, 46022 Valencia, Spain; 4 Instituto Agroforestal Mediterráneo, Universitat Politècnica de València, Camino de Vera 14, 46022 Valencia, Spain

**Keywords:** Genetics, Genome

## Abstract

The wild *Solanum pimpinellifolium* (SP) and the weedy *S. lycopersicum* var. *cerasiforme* (SLC) are largely unexploited genetic reservoirs easily accessible to breeders, as they are fully cross-compatible with cultivated tomato (*S. lycopersicum* var. *lycopersicum*). We performed a comprehensive morphological and genomic characterization of four wild SP and four weedy SLC accessions, selected to maximize the range of variation of both taxa. These eight accessions are the founders of the first tomato interspecific multi-parent advanced generation inter-cross (MAGIC) population. The morphoagronomic characterization was carried out with 39 descriptors to assess plant, inflorescence, fruit and agronomic traits, revealing the broad range of diversity captured. Part of the morphological variation observed in SP was likely associated to the adaptation of the accessions to different environments, while in the case of SLC to both human activity and adaptation to the environment. Whole-genome resequencing of the eight accessions revealed over 12 million variants, ranging from 1.2 to 1.9 million variants in SLC and from 3.1 to 4.8 million in SP, being 46.3% of them (4,897,803) private variants. The genetic principal component analysis also confirmed the high diversity of SP and the complex evolutionary history of SLC. This was also reflected in the analysis of the potential footprint of common ancestors or old introgressions identified within and between the two taxa. The functional characterization of the variants revealed a significative enrichment of GO terms related to changes in cell walls that would have been negatively selected during domestication and breeding. The comprehensive morphoagronomic and genetic characterization of these accessions will be of great relevance for the genetic analysis of the first interspecific MAGIC population of tomato and provides valuable knowledge and tools to the tomato community for genetic and genomic studies and for breeding purposes.

## Introduction

Tomato (*Solanum lycopersicum* L.) is the most important vegetable crop with a global production of 182 million tons per year, 28.6% more than in the previous decade^1^. Tomato belongs to the Solanaceae family, genus *Solanum* L., section Lycopersicon^2^ and is generally divided into two subspecific taxa, corresponding to botanical varieties: the cultivated variety with big-sized fruits, *S. lycopersicum* var. *lycopersicum* (SLL), and the weedy with small-sized fruits *S. lycopersicum* var. *cerasiforme* (SLC). The closest wild relative of SLL is *S. pimpinellifolium* L. (SP)^2,3^. *Solanum lycopersicum* var. *cerasiforme* has been recognized as the ancestor of the cultivated tomato. A two-step model of domestication in which a pre-domestication took place in the Andean region, with the domestication being completed in Mesoamerica, has been proposed^4,5^. The most ancestral SLC originated from Peruvian and Ecuadorian SP, and a considerable bottleneck took place when SLC migrated from those regions to Mesoamerica. More recently Razifard et al.^6^ suggested that the evolutionary history was more complex. In any case, an even more severe bottleneck was produced when SLL was imported to Europe^5,7^. Therefore, these studies point to Ecuadorian and Peruvian SP and SLC as a barely exploited reservoir of genetic diversity. Recently, a comprehensive study has been conducted by analysing the resequencing data of 725 phylogenetically and geographically representative accessions that included mainly the two varieties of *S. lycopersicum* and the wild relative *S. pimpinellifolium*^7^. The already known important narrowing of the genetic basis of the cultivated tomato due to the domestication process^5,8^ has been corroborated in this study^7^. This important loss of genes, estimated at around 200, was relevant even between accessions of *S. pimpinellifolium* from Peru and the ones growing in the coastal region of northern Ecuador, suggesting an adaptation of these accessions to their specific environments when spreading from the centre of origin to the North. Enrichment analysis indicated that defence response was the most important category of genes lost during domestication, particularly for genes related to cell-wall thickening, which influences biotic and abiotic stresses through fortification of the cell wall^7^. This may contribute to explain the extreme susceptibility of cultivated tomato to pests and diseases, as well as the lack of adaptation to unfavourable environmental conditions.

*Solanum pimpinellifolium* (SP) inhabits the coastal regions of Ecuador, Peru and northern Chile. The natural range of this species includes such contrasting environments as the northern coastal Ecuadorian tropical rainforests and the Peruvian coastal desert^9^. Peruvian and Ecuadorian accessions have been found to be genetically differentiated^10^, with an important correlation between genetic differentiation and climate. This species, fully cross-compatible with tomato^11^ and with such a wide range of distribution, is a potential source of genes of interest for resistance to diseases and tolerance to abiotic stresses. In fact, several genes of resistance have been identified in this species and introgressed in most of the commercial hybrids^12–14^. In addition, adaptation to abiotic stresses such as salinity and water deficit has also been found^15^. The detailed study and annotation of its genome, recently published^16^, confirms that this species offers a wealth of breeding potential for desirable traits and displays an enrichment in genes involved in biotic and abiotic stresses responses. Although this species has been used for tomato breeding, its wide area of distribution and the marked genetic differentiation of the different populations according to their geographic distribution, suggest that a wealth of useful diversity has not yet been explored in breeding.

*Solanum lycopersicum* var. *cerasiforme* grows spontaneously worldwide in tropical and subtropical regions^17^. It has been collected in a wide range of habitats that includes deserts and very humid regions in altitudes that range from sea level up to 2,400 m^9^. It is widely distributed close to human-modified areas, such as home gardens and orchards, usually without human intervention, behaving as a weed. SLC has been much less exploited in breeding than SP. However, its adaptation to humid areas is of special interest, as resistance to some pathogens such as *Oidium lycopersici*^18^ and to *Phytophthora infestans*^19^ has been described.

In this study, we present a comprehensive morphological phenotyping and whole-genome resequencing, along with an extensive structural and functional characterization, of four SP and four SLC accessions. These highly diverse tomato relatives were selected from over 1,000 tomato accessions, genotyped with 7,720 SNPs^5^, to maximize variation for origin, morphology and genetic diversity for developing the first interspecific multi-parent advanced generation inter-cross (MAGIC) population in tomato. These MAGIC founders have already demonstrated to be resistant and tolerant to several abiotic and biotic stresses^20^.

The aim of this study is to interrogate these accessions to provide valuable information regarding variation for traits of interest and which of those will be segregating in the forthcoming MAGIC population, as well as to provide a large set of robust variants to efficiently perform the genetic dissection of traits of interest. Likewise, the information developed in this study will be of interest for tomato breeders, for further genetic and genomic studies involved in tomato domestication, and to increase the precision and accuracy of future tomato pan-genomes.

## Material and methods

### Plant material

**Fig. 1:**
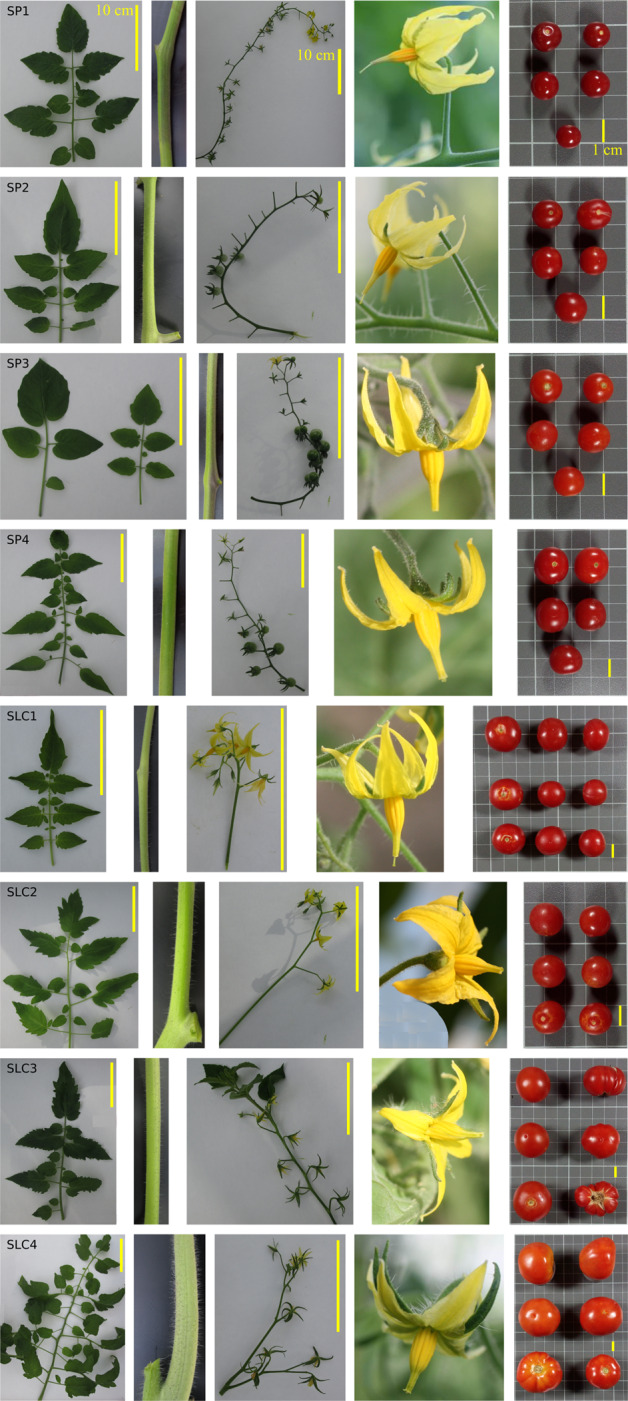
Pictures of leaves, stem, inflorescence, flower and fruit of the four *S. pimpinellifolium* (SP1–SP4) and four *S. lycopersicum* var. *cerasiforme* (SLC1–SLC4) accessions used in this study. The yellow bars in the upper right of leaves and inflorescence pictures indicate 10 cm, while the yellow bars in the lower right of fruit pictures indicate 1 cm. The grid cells in fruit pictures have a size of 1 cm × 1 cm

Four SP (SP1–SP4) and four SLC (SLC1–SLC4) accessions were selected from Blanca et al.^5^ to maximize the genetic and phenotypic diversity, as well as different origins, of these tomato relatives. Accessions were provided by the genebank of COMAV, Universitat Politècnica de València, Spain (BGV codes), the Tomato Genetic Resources Center (University of California, Davis) and the Agricultural Research Service, USDA, (Table 1). The selected materials encompass a wide range of variation concerning vegetative, plant and fruit morphology traits, adaptation to different environments and biotic and abiotic stresses, and together capture an important fraction of the genetic diversity of tomato^5^ (Fig. 1). The segregation and variability for morphological traits is evident in the S3 generation of the MAGIC population that is under development (Supplementary Data S1).

**Table 1:** Collection sites and GPS coordinates of the four *S. pimpinellifolium* (SP) and four *S. lycopersicum* var. *cerasiforme* (SLC) used in this study.

	*S. pimpinellifolium*				*S. lycopersicum* var. *cerasiforme*			
Accession code	BGV006454	BGV015382	BGV013720	BGV007145	BGV006769	BGV007931	LA2251	PI487625
Study code	SP1	SP2	SP3	SP4	SLC1	SLC2	SLC3	SLC4
Collection site	Chulucanas, Piura, Peru	Jamalca, Department of Amazonas, Peru	Nazca, Ica, Peru	El Carmen, Manabí, Ecuador	Cayambe Coca National Park, Napo, Ecuador	Ahome, Sinaloa, Mexico	Soritor, San Martín, Peru	Los Diamantes, Costa Rica
Latitude	5°08′51″S	5°53′08″S	14°34′49″S	0°13′19″S	0°01′52″S	26°03′00″N	6°08′00″S	9°60′61″N
Longitude	80°16′13″W	78°10′34″W	74°53′14″W	79°29′20″W	77°47′10″W	109°22′00″W	77° 05′ 00″ W	84°14′03″W

### Phenotyping trial

Ten plantlets per accession (*n* = 10) were produced in a commercial nursery and transplanted at 6–7 true-leaves stage to a mesh greenhouse in El Perelló, Valencia, Spain (GPS coordinates: latitude, 39°16′38″N; longitude, 0°16′37″W; 3 m above sea level). Plants were grown on a loamy sand soil, irrigated and fertilized using drip irrigation system, pruned at one stem and trained with vertical strings. The accessions were phenotyped for 39 morphoagronomic traits^21^, corresponding to plant (10), inflorescence and flower (5), fruit (18), internal fruit quality (4) and agronomic value (2) descriptors (Table 2).

**Table 2:** List of the 39 traits with abbreviations and units used for the morphoagronomic characterization of the four *S. pimpinellifolium* (SP) and four *S. lycopersicum* var. *cerasiforme* (SLC) accessions of this study.

Code	Descriptor name	Descriptor scale/unit
Plant descriptors		
SPD	Stem pubescence density	1–7 (1: absent; 7: dense)
SA	Stem anthocyanin coloration	0–2 (0: absent; 2: high)
LT	Leaf type	1–6 (1: dwarf; 2: potato; 3: standard; 4: *S. peruvianum*; 5: *S. pimpinellifolium*; 6: other)
LN	Leaflets number	Number
SLN	Small leaflets number	Number
LB	Leaflet border	1–4 (1: entire; 4: highly serrated)
LVA	Leaf vein anthocyanin presence	0–1 (0: absent; 1: present)
1TH	1st truss height	cm
3TL	3rd truss length	cm
5TL	5th truss length	cm
Inflorescence descriptors		
IT	Inflorescence type	1–3 (1: uniparous; 2: both; 3: multiparous)
FPT	Flowers per truss (2nd and 3rd)	Number
LP	Presence of leaves in the inflorescence	0–2 (0: absent; 2: presence in all)
SP	Presence of shoots in the inflorescence	0–2 (0: absent; 2: presence in all)
SPOS	Style position	0–7 (0: inserted; 7: highly exerted)
Fruit descriptors		
FC	Exterior colour of immature fruit	1–9 (1: greenish-white; 9: very dark green)
GI	Greenback intensity	0–7 (0: absent; 7: strong)
FP	Fruit pubescence	0–7 (0: absent; 7: dense)
FS	Fruit shape	1–4 (1: flattened; 2: slightly flattened; 3: round; 4: elongated)
RCE	Ribbing at calyx end	1–7 (1: very weak; 7: strong)
FCS	Fruit cross-sectional shape	1–3 (1: round; 3: irregular)
SPS	Shape of pistil scar	1–4 (1: dot; 2: stellate; 3: lineal; 4: irregular)
BES	Fruit blossom end shape	1–3 (1: indented; 4: pointed)
FF	Fruit fasciation	3–7 (3: slight; 7: severe)
FH	Fruit height	mm
FW	Fruit width	mm
W	Fruit weight	g
SS	Shoulder shape	1–7 (1: flat; 7: strongly depressed)
PT	Pericarp thickness	mm
LOC	Locule number	Number
L	Exterior fruit colour lightness	L
A	Exterior fruit colour a	a
B	Exterior fruit colour b	b
Internal fruit quality descriptors		
Acid	Acidity	% of citric acid (1 g/sample)
Brix	Sugar content	°Brix
AA	Ascorbic acid	mg/L (liquid extract)
pH	pH	
Agronomic descriptors		
DM	Days to maturity of first fruit^a^	Number of days
D50%	Days until 50% of plants with mature fruits^b^	Number of days

^a^From sowing until one plant has one ripe fruit

^b^From sowing until 50% of plants have at least one fruit ripened

Vegetative, flower and fruit traits were recorded once per plant (*n* = 10) in fully developed plants presenting fully ripen fruits in the first and second trusses. In addition, fruit length, fruit width, fruit weight (FW), CIE L*a*b* 1976 colour space coordinates, pericarp thickness and locule number (LOC) were measured in five representative fruits per plant (*n* = 50) from which a mean value per plant was calculated. Finally, between 5 and 15 fruits per plant (*n* = 10), randomly collected from the second, third and fourth trusses, were grinded together and the resulting juice used to assess pH, acidity (Acid), °Brix and ascorbic acid (AA) content. Individual plant values (*n* = 10) were used to calculate accession means regarding the 39 evaluated traits for subsequent multivariate data analysis. In addition, species mean values for each trait were obtained from the average of the four accessions belonging to the species. Finally, Student’s *t* tests were performed in order to detect significant differences between the means of both taxa for quantitative traits. For that, the function “t.test” of the R package stats^22^ (v3.6.1) was used with a confidence level of 95%. Numeric differences of the data matrix were visualized by a hierarchical clustering heatmap using ClustVis^23^. Principal component analysis (PCA) was performed using the function “prcomp” of the R package stats^22^ (v3.6.1) and plotted with “ggplot2”^24^.

### Sequencing

Genomic DNA was extracted from 100 mg of fresh young leaves following an in-house protocol specifically developed for sequencing applications^23^. DNA integrity was checked by electrophoresis on 0.8% agarose gel and by spectrophotometry using a NanoDrop™ ND-1000 (Thermo Fisher Scientific, Waltham, MA, USA) spectrophotometer checking the A260/A280 and A260/A230 ratios. DNA quantity was measured by fluorometry using a Qubit^®^ 2.0 (Thermo Fisher Scientific, Waltham, MA, USA) fluorometer. High-quality DNA samples were shipped to the CNAG-CRG research centre (Barcelona, Spain) for libraries construction and sequencing. Paired-end libraries were prepared with NO-PCR protocol using KAPA Library Preparation kit (Roche, Basel, Switzerland) using 2 µg of genomic DNA, sheared on a Covaris™ LE220 (Covaris Inc., Woburn, MA, USA) focused ultrasonicator in order to reach the fragment size of ~500 bp. Fragmented genomic DNA was then end-repaired, adenylated and ligated to Illumina platform compatible adaptors with dual indexes (Integrated DNA Technologies, Coralville, IA, USA). The adaptor-modified end library was size selected and purified with AMPure XP beads (Beckman Coulter, Brea, CA, USA). Final libraries were quantified by Kapa Library Quantification Kit for Illumina platforms (Kapa Biosystems, Wilmington, MA, USA). Finally, libraries were sequenced on two lines of an Illumina HiSeq4000 (Illumina Inc., San Diego, CA, USA) sequencing platform with a read length of 300 bp.

### Reads processing, mapping and variant calling

Ea-utils were used to process the raw reads, using the tool fastq-mcf to remove sequencing adaptors, reads shorter than 50 bp, or with a Phred score lower than 30. Finally, the tool fastq-stats was used to perform basic stats^25^. High-quality reads were then mapped to Heinz 1706 tomato reference genome (version SL4.0)^26^ using the Bowtie2 aligner set to default parameters^27^. Samtools were used to convert, filter and make stats of the mapped reads^28^, while mapping coverage was calculated with the genomecov utility of the BEDtools package^29^, and the average coverage with an in-house script. Variants were called using FreeBayes^30^ (v1.3) with minimum depth coverage of 5, minimum base quality of 20, and minimum mapping quality of 20, while missing data were removed with VCFtools^31^ (0.1.15). To recognize similar variant distribution patterns among samples, variants were divided into 10 Kb bins using GenoToolBox utilities (https://github.com/aubombarely/GenoToolBox) and plotted in R^32^. PCA was performed using R packages vcfR^33^, Adegenet^34^ and ggplot2^35^ with the whole set of SNPs, after removing the rest of variant types, missing data and chromosome 0.

### Variants and genome annotation

Variant effects were estimated using the SnpEff software^36^ (version 4.2) and classified by impact, functional class, type and region affected, along with the annotation of substitution mutations, amino acids replacement and codon changes. GO terms from the GO database (http://www.geneontology.org/) were associated with those variants predicted as “high” impact using the tomato reference genome gff3 annotation file, the R package topGO^37^ and an in-house R script. Significant terms were plotted using REVIGO^38^. The variants identified in some of the genes that might control the morphoagronomic traits assessed in this study were filtered from the VCF using an in-house script. The detailed information about the candidate genes was retrieved from the Sol Genomics Network database (https://solgenomics.net/).

### Data access

The raw data have been deposited into the NCBI Short Read Archive under submission identifier SUB7195326 with the Bioproject identifier PRJNA616074. Accessions are indexed with BioSample IDs from SAMN14480924 to SAMN14480931. VCF files with the corresponding variants identified are available upon request to the corresponding author.

## Results

### Morphoagronomic characterization

The eight accessions tested displayed a great variation for most of the recorded traits (Supplementary Data S2). Regarding vegetative traits, SP accessions exhibited higher variation than those of SLC, particularly for stem anthocyanin (SA) coloration, which was very intense in SP3, small leaflets number (SLN) and the length of the 3rd (3TL) and 5th (5TL) inflorescences. However, for other inflorescence traits, the accessions of SLC were more variable. SLC accessions displayed different types of inflorescences, ranging from uniparous to irregular ones and often presenting leaves and shoots at the truss’ terminal end. This contrasted with the generally uniparous and long inflorescences found in SP, although the number of flowers varied greatly within this species, ranging from the 10 flowers in the accession SP4 from Ecuador to 20 in SP1 from Peru. Furthermore, the style position (SPOS) was considerably more exerted in SP, but with considerable variation, ranging from the stigma being at the same level of the anthers cone in the accessions from Ecuador, to highly exerted stigmas in the accessions coming from Peru. Regarding fruit traits, there was higher variation in SLC regarding size (fruit height, FH, and FW), ribbing (RCE), shape of pistil scar (SPS) and LOC. However, the b parameter of the CIE L*a*b* 1976 colour space, which measures blue–yellow component, was more variable in SP. The same occurred for other traits related to fruit internal quality, such as Acid, °Brix and ascorbic acid AA. Finally, the variation observed for ripening earliness (DM and DM50%) was similar among the accessions within each species. When comparing the ranges of the traits differentiating both species, only the leaf traits LT and LB, fruit traits FH and PT, and the ripening earliness showed non-overlapping ranges between both species (Table 3). The wide range of variation found among the accessions, masked the differences between both species, especially in SLC accessions where the high morphological variability of this variety was remarkable for FW, ranging from the 1.91 to 23.83 g.

**Table 3:** Mean and range for the groups of four *S. pimpinellifolium* and four *S. lycopersicum* var. *cerasiforme* for the morphoagronomic descriptors assessed in this study.

Code	*S. pimpinellifolium*		*S. lycopersicum* var. *cerasiforme*	
	Mean	Range	Mean	Range
Plant descriptors				
SPD	2.30	0.00–5.00	5.67	3.00–7.00
SA	1.14	0.56–2.00	0.35	0.00–1.00
LT	3.00	3.00–3.00	5.00	5.00–5.00
LN	6.74	6.40–7.00	7.37	6.67–8.80
SLN	8.24	3.15–17.00	13.33	8.00–17.56
LB	1.54	1.00–2.00	2.79	2.17–3.00
1TH	10.92	5.50–17.00	13.98	8.00–17.72
3TL	20.57	11.35–28.44	16.39	10.75–19.75
5TL	26.12	13.06–36.17	21.03	17.25–30.17
Inflorescence descriptors				
IT	1.15	1.00–1.60	1.63	1.00–2.00
FPT	15.84	10.00–20.67	9.41	8.40–11.33
LP	0.59	0.00–1.00	0.95	0.33–1.56
SP	0.23	0.00–0.60	0.89	0.00–1.78
SPOS	5.00	3.00–7.00	2.75	0.00–5.00
Fruit descriptors				
FC	2.00	1.00–3.00	1.50	1.00–3.00
GI	3.50	3.00–5.00	3.83	3.00–6.00
FP	2.25	0.00–3.00	3.88	3.00–5.00
FS	2.75	2.00–3.00	2.46	1.45–3.00
RCE	0.00	0.00–0.00	0.79	0.00–1.67
FCS	1.00	1.00–1.00	1.42	1.00–2.00
SPS	1.00	1.00–1.00	1.10	1.00–1.39
BES	2.00	2.00–2.00	2.00	2.00–2.00
FF	0.00	0.00–0.00	0.72	0.00–1.67
FH	1.51	1.46–1.60	2.24	1.73–2.80
FW	1.34	1.07–1.76	2.49	1.47–3.70
W	1.52	0.79–2.89	10.39	1.91–23.83
SS	1.50	1.00–3.00	2.08	1.00–5.00
PT	0.17	0.12–0.20	0.30	0.18–0.39
LOC	2.00	2.00–2.00	2.35	2.00–3.04
L	31.27	29.42–35.13	32.21	30.12–35.98
A	27.09	22.46–29.63	21.47	16.47–24.59
B	11.96	9.41–16.52	12.45	10.62–15.04
Internal fruit quality descriptors				
Acid	1.00	0.75–1.43	0.72	0.40–0.90
Brix	5.37	3.15–6.83	4.69	3.15–5.89
AA	30.77	23.00–43.10	32.03	26.53–40.67
pH	4.26	4.09–4.46	4.31	4.01–4.56
Agronomic descriptors				
DM	55.00	52.00–58.00	66.67	62.00–74.00
DM50%	56.25	55.00–58.00	69.63	65.00–75.00

**Fig. 2:**
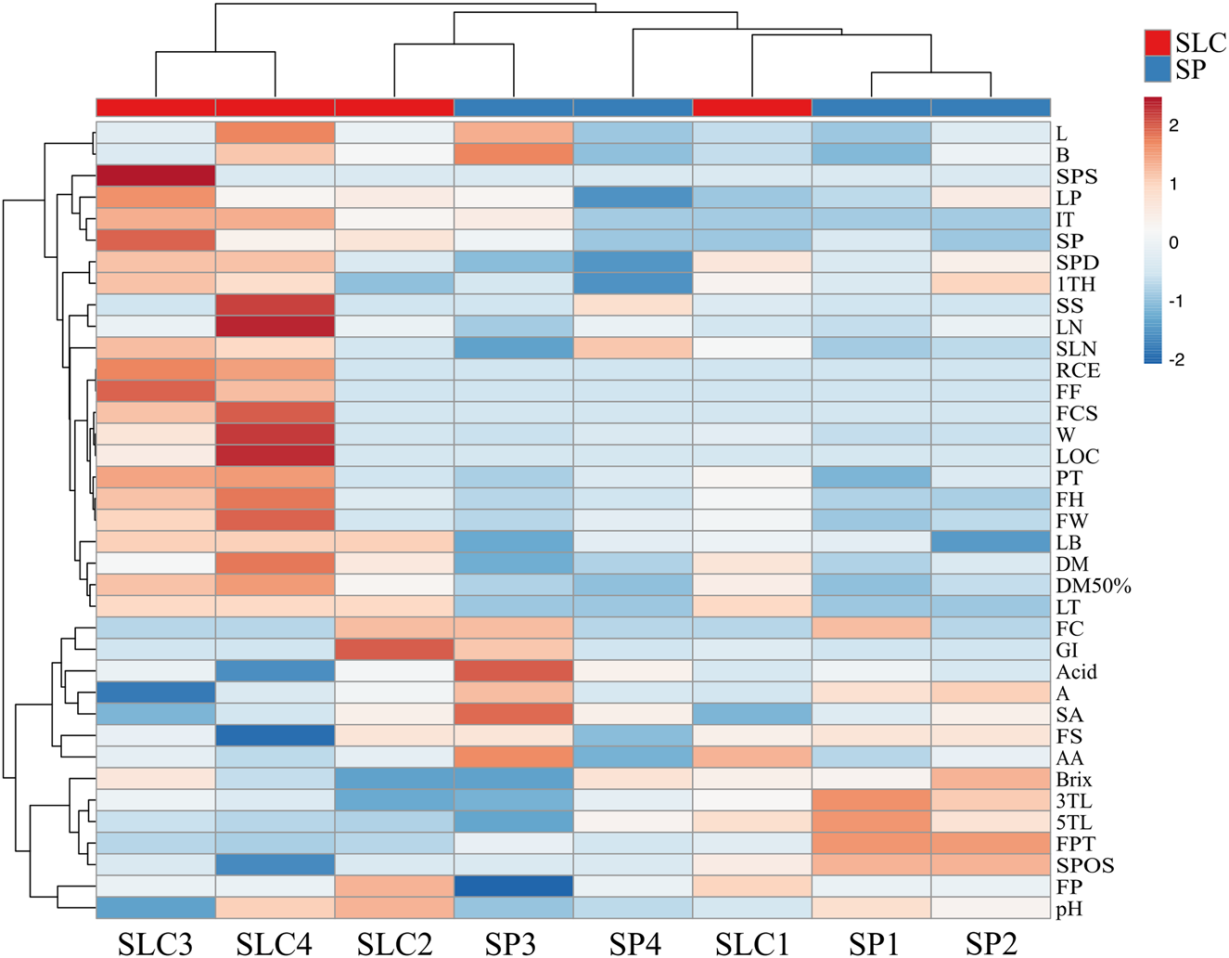
Correlations among morphoagronomic traits and accessions. Hierarchical clustering heatmap for the four *S. pimpinellifolium* (SP1–SP4) and *S. lycopersicum* var. *cerasiforme* (SLC1–SLC4) accessions for the 37 polymorphic morphoagronomic traits assessed in this study

The hierarchical clustering heatmap showed the correlations among the recorded traits, as well as among the accessions studied (Fig. 2 and Supplementary Data S3). The traits recorded can be divided into two clusters, one formed by 23 traits and the other by 14 traits. Furthermore, the first cluster can be divided in two subclusters, one of them including 13 traits and the other one 11. The first subcluster included traits related to fruit size (RCE, FE, FCS, W, LOC, PT, FH, FW), highly correlated among them, which displayed high values for SLC3 and SLC4, the two accessions of SLC bearing bigger fruits. These traits showed also high correlations with others related to leaf (LB, LT) and ripening earliness (DM, DM50%), indicating that these accessions of SLC have more complex leaves and that they need more days to reach fruit maturity. The 11 traits of the other subgroup were also highly correlated with SL3 and SL4 accessions and were, in general, related with stem pubescence density (SPD), the height of the 1st truss (1TH), leaves (LN, SLN), inflorescence (IT, SPH, LP) and fruit (L, B, SPS, SS). The second group of 14 traits included five of the internal fruit quality traits (Acid, AA, °Brix and pH), fruit colour (FC, GI, A) and others related to the inflorescence and flower (3TL, 5TL, FPT, SPOS). This group of traits showed higher values in three of the SP accessions (SP1, SP2 and SP4), although with some differences due to the great morphological variability found in this species. SP3 was the accession most differentiated from the rest of SP accessions for these traits.

Finally, PCA was performed to study the relationship among traits and accessions used herein. The first and second components (PC) accounted for 47.0% and 19.1% of the total variation, respectively. The PC1 was positively correlated with FW and size traits (FH and W) and other fruit morphological characteristics (PT, FCS, RCE, LOC and FF) (Supplementary Data S4). Leaf traits (LT, LN, SLN and LB), as well as the type of inflorescence (IT) and the ripening earliness (DM, D50%) were also positively correlated with PC1. The number of FPT, the SPOS, the fruit shape (FS) and the colour coordinate a (green–red component), were the ones with lowest (negative) values for this PC1. Regarding PC2, the length of the inflorescences (3TL and 5TL) and the °Brix were the traits with the highest positive contribution, whereas SA coloration, the colour of immature fruit (FC), Acid and luminosity (L) were the traits with higher negative contribution. The projection of the accessions on a two-dimensional PCA plot resulted in a distribution congruent with their morphological characteristics (Fig. 4a). The accessions SLC3 and SLC4 located at the right part of the plot. These accessions are the most phenotypically similar to the cultivated tomato, bearing bigger, slightly flattened and occasionally ribbed fruits, different from the commonly round and small fruits of this taxon. The accession SLC1 grouped with some of the SP accessions due its intermediate characteristics between both species. SLC2 was also located separately from the other SLC accessions. In this case, the small size of its fruits, like those of SP, was the main reason for the separation from the other SLC accessions. Three SP accessions, SP1, SP2 and SP4 grouped together at the top left part of the plot due to their tiny fruits, high stigmatic exertion, higher number of fruits per truss and ripening earliness. The traits responsible for the separation of SP3 from the rest of the SP accessions were the SA coloration, trait highly negatively correlated with the PC2, as well as the darker colour of its immature fruits and its highly acidic fruits.

### Whole-genome sequencing

The sequencing of the eight accessions generated over 0.9 billion 150 bp paired-end raw reads (137.7 Gb), averaging 114.2 million reads per sample (Table 4). After the cleaning step, an average of 96.0% high-quality reads was mapped to the Heinz 1706 tomato reference genome (version SL4.0)^26^, with an average depth of coverage that varied from 17.0-fold for SP4 to 21.0-fold for SLC1. As expected, the average coverage of the reference genome was higher for SLC accessions (averaging 98.4%), than for SP ones (96.7%). The average depth of mapping coverage and the average coverage of the reference genome across the chromosomes is detailed in Supplementary Data S5.

**Table 4:** Statistics of sequencing and mapping of the four *S. pimpinellifolium* and four *S. lycopersicum* var. *cerasiforme* accessions re-sequenced using Heinz 1706 SL4.0 as a reference genome^26^ (RF).

	*S. pimpinellifolium*				*S. lycopersicum* var. *cerasiforme*				Mean	Total
	SP1	SP2	SP3	SP4	SLC1	SLC2	SLC3	SLC4		
Raw reads (million)	122.7	105.4	108.3	105.1	124.0	112.1	119.8	116.9	114.2	914.3
Yield (Gb)	18.5	15.9	16.3	15.8	18.7	16.9	18.0	17.6	17.2	137.7
High-quality reads (million)	122.3	105.3	108.3	105.0	123.9	112.1	119.7	116.8	114.1	913.4
Total nt. (billion)	18	16	16	16	19	17	17.9	17	17.0	136.4
Reads mapped (million)	116	100	102	101	120	110	116	114	109.8	879.1
% reads mapped	94.4	95.1	94.3	95.9	96.7	97.3	97.1	97.3	96.0	–
Average depth of coverage	20.2	17.1	18.0	17.0	21.0	18.5	19.0	19.4	18.7	–
% average coverage RF	96.8	96.8	95.5	97.0	98.1	98.7	98.7	98.2	97.4	–

### Variant calling, polymorphism distribution and genetic relationships analysis

Among the eight accessions, a total of 12,833,972 variants were detected when compared with the reference genome. However, in order to keep only the most reliable variants for genetic and genomic studies and breeding purposes, the variants detected in chromosome 0 and those that presented missing data were removed, yielding a total of 10,557,258 high-quality variants (Table 5). Most of those variants were SNPs (9,106,964; 86.2%), followed by InDels (812,034; 7.7%), complex variations (579,456; 5.5%) and multiple-nucleotide polymorphisms (MNPs, 58,804; 0.6%). The total variants in SLC accessions, ranged from 1.2 in SLC2 to 1.9 million in SLC1, while that of the SP accessions ranged from 3.1 in SP4 to 4.8 million in SP3. All accessions displayed higher proportion of homozygous variants than heterozygous ones. Surprisingly, SP accessions, except SP1, showed a lower heterozygous variants percentage, from 5.4% to 7.3% (with an average of 9.0% including SP1), than SLC accessions, from 8.2% to 13.1% (with an average of 11.3%). However, the percentage of heterozygous variants with the reference genome was slightly higher in SP accessions, with an average of 0.049%, than in SLC ones with an average of 0.022%. The number of private variants (i.e. accession specific) was 4,897,803 (46.3% of the total subset) and variable in both subsets, ranging from 14.5% in SLC3 to 30.9% in SP4.

**Table 5:** Summary of the variants identified in the four *S. pimpinellifolium* and four *S. lycopersicum* var. *cerasiforme* accessions using using Heinz 1706 SL4.0 as a reference genome^26^ (RF).

	*S. pimpinellifolium*										*S. lycopersicum* var. *cerasiforme*										Total	
	SP1		SP2		SP3		SP4		Mean		SLC1		SLC2		SLC3		SLC4		Mean			
	Count	%	Count	%	Count	%	Count	%	Mean	%	Count	%	Count	%	Count	%	Count	%	Count	%	Count	%
Variants																						
Homozygous SNPs	3,223,730	69.5	3,032,979	78.5	3,879,974	79.4	2,453,673	76.9	3,147,589	76.1	1,497,288	76.0	855,877	68.9	912,198	69.3	1,115,582	72.2	1,095,236	71.6	9,106,964	86.2
Heterozygous SNPs	647,201	14.0	184,801	4.8	209,810	4.3	189,876	6.0	307,922	7.3	129,834	6.6	125,920	10.1	143,599	10.9	149,794	9.7	137,287	9.3		
Homozygous InDels	379,109	8.2	381,565	9.9	447,678	9.2	324,948	10.2	383,325	9.4	217,208	11.1	172,779	13.9	161,126	12.2	175,312	11.3	181,606	12.1	812,034	7.7
Heterozygous InDels	84,534	1.8	18,288	0.4	20,876	0.4	16,667	0.5	35,091	0.8	11,262	0.6	9,928	0.8	9,689	0.7	10,972	0.7	10,463	0.7		
Homozygous complex	209,751	4.5	188,390	4.8	258,072	5.3	151,032	4.8	201,811	4.9	76,890	3.9	53,003	4.2	60,497	4.6	61,577	4.0	62,992	4.2	579,456	5.5
Heterozygous complex	57,559	1.2	26,997	0.7	32,071	0.6	24,840	0.8	35,367	0.8	18,838	0.9	15,018	1.2	18,388	1.4	18,824	1.2	17,767	1.2		
Homozygous MNP	32,803	0.7	29,863	0.8	36,797	0.7	23,648	0.7	30,778	0.7	15,916	0.8	9,748	0.8	11,157	0.8	12,513	0.8	12,334	0.8	58,804	0.6
Heterozygous MNP	4906	0.1	2144	0.1	2469	0.1	1937	0.1	2864	0.1	1501	0.1	1,055	0.1	1,261	0.1	1,355	0.1	1,293	0.1		
Total homozygous	3,845,393	82.9	3,632,797	94.0	4,622,521	94.6	2,953,301	92.7	3,763,503	91.1	1,807,302	91.8	1,091,407	87.8	1,144,978	86.9	1,364,984	88.3	1,352,168	88.7	–	–
Total heterozygous	794,200	17.1	232,230	6.0	265,226	5.4	233,320	7.3	381,244	9.0	161,435	8.2	151,921	12.2	172,937	13.1	180,945	11.7	166,810	11.3	–	–
Total variants	4,639,593	100.0	3,865,027	100	4,887,747	100.0	3,186,621	100.0	4,144,747	100.0	1,968,737	100.0	1,243,328	100.0	1,317,915	100.0	1,545,929	100.0	1,518,977	100.0	10,557,258	100.0
% het. variants with RF	0.103		0.030		0.034		0.030		0.049		0.021		0.020		0.022		0.023		0.022			
% total variants with RF	0.600		0.500		0.632		0.412		0.536		0.255		0.161		0.171		0.200		0.197			
Private variants	771,181	16.6	748,354	19.3	1,115,616	22.8	985,453	30.9	905,151	22.4	339,481	17.2	467,864	37.6	190,559	14.5	279,295	18.0	319,299.8	21.8	4,897,803	46.3
Variants in coding regions																						
Homozygous SNPs	307,824	58.6	321,285	71.8	375,090	72.3	253,723	69.1	314,481	68.0	124,716	65.0	92,841	60.0	78,951	56.0	80,019	55.2	94,132	59.1	996,772	82.4
Heterozygous SNPs	107,212	20.4	30,776	6.9	32,981	6.4	34,522	9.4	51,373	10.8	22,275	11.6	23,504	15.2	27,654	19.6	30,294	20.9	25,932	16.8		
Homozygous InDels	58,463	11.1	61,945	13.9	70,754	13.7	51,374	14.0	60,634	13.2	29,821	15.6	26,057	16.9	22,305	15.8	22,568	15.5	25,188	15.9	131,056	10.8
Heterozygous InDels	16,675	3.2	3073	0.7	3411	0.6	2899	0.8	6515	1.3	1802	0.9	1,719	1.1	1,612	1.1	1,880	1.3	1,753	1.1		
Homozygous complex	22,053	4.2	22,772	5.0	27,306	5.3	17,461	4.8	22,398	4.8	8323	4.3	6,548	4.2	5,826	4.1	5,476	3.8	6,543	4.1	76,511	6.3
Heterozygous complex	10,199	1.9	4550	1.0	5509	1.0	4551	1.2	6202	1.3	3326	1.7	2,864	1.9	3,763	2.7	3,835	2.6	3,447	2.2		
Homozygous MNP	2831	0.5	2816	0.6	3228	0.6	2206	0.6	2770	0.6	1282	0.7	947	0.6	849	0.6	872	0.6	988	0.6	5,909	0.5
Heterozygous MNP	749	0.1	327	0.1	342	0.1	315	0.1	433	0.1	220	0.1	170	0.1	212	0.1	203	0.1	201	0.1		
Total homozygous	391,171	74.3	408,818	91.3	476,378	91.8	324,764	88.5	400,283	86.5	164,142	85.6	126,393	81.7	107,931	76.4	108,935	75.0	126,850	79.7	–	–
Total heterozygous	134,835	25.7	38,726	8.7	42,243	8.2	42,287	11.5	64,523	13.5	27,623	14.4	28,257	18.3	33,241	23.6	36,212	25.0	31,333	20.3	–	–
Total variants	526,006	100.0	447,544	100.0	518,621	100.0	367,051	100.0	464,806	100.0	191,765	100.0	154,650	100.0	141,172	100.0	145,147	100.0	158,184	100.0	1,210,248	100.0
% het. variants with RF	0.017		0.005		0.005		0.005		0.008		0.003		0.004		0.004		0.005		0.004			
% variants with RF	0.068		0.058		0.067		0.047		0.060		0.024		0.020		0.018		0.019		0.020			

The total variants identified in coding regions were 1.2 million (11.4% of the total), being 82.4% of them SNPs, 10.8% InDels, 6.3% complex variations and 0.5% MNPs, and the average number of variants with respect to the total set in SP accessions was 3.1-fold higher than those in SLC accessions. All accessions displayed higher percentage of heterozygous variants in coding regions compared to the variants in the whole genome (Table 5).

The average distribution of the variants among the chromosomes displayed considerable differences; over 3.5-fold between chromosome 11 (56,163 variants) and chromosome 8 (197,314) in SLC and 1.7-fold between chromosome 11 (279,074) and chromosome 1 (486,526) in SP accessions (Supplementary Data S6). Furthermore, even greater variation was observed among the accessions for the same chromosome, especially for the SLC set, with differences over eightfold in chromosome 7 and fivefold in chromosomes 2, 3, 5 and 9, even though the total variants only differed 1.5-fold on average. Obviously, this variation was also reflected in the variant rate, ranging in SP accessions from one variation every 378 bp in chromosome 8 in SP4 to one every 121 bp in the same chromosome in SP1 (with an average value over the genome of one every 186 bp), and one variant every 2,309 bp in chromosome 7 to one every 143 bp in chromosome 8 in SLC3 for SLC accessions (with an average of one variant every 509 bp) (Supplementary Data S6).

**Fig. 3:**
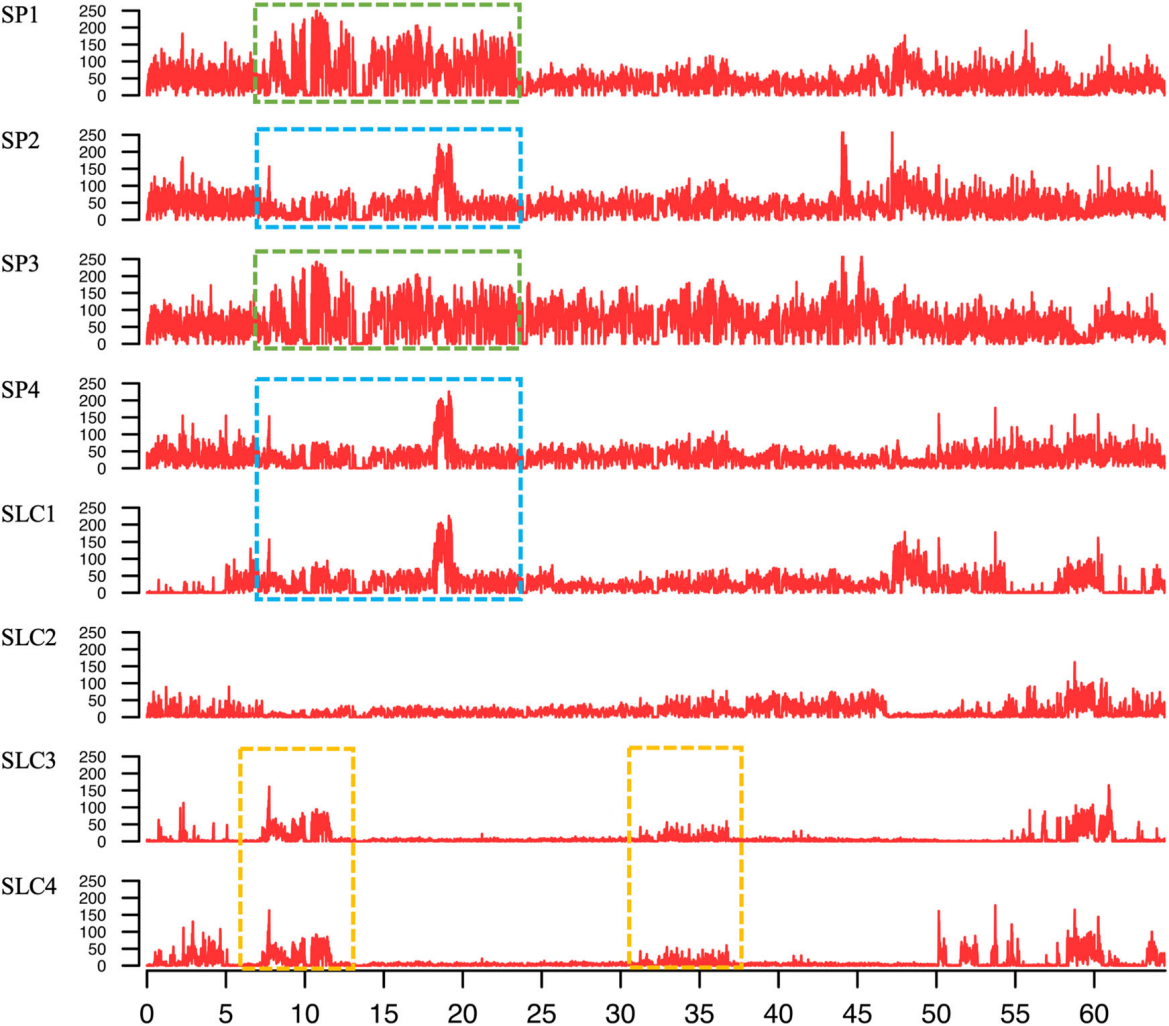
Distribution of homozygous variants along chromosome 4 for the four *S. pimpinellifolium* (SP1–SP4) and four *S. lycopersicum* var. *cerasiforme* (SLC1–SLC4) accessions. The peaks represent high frequencies of variants in a window size of 10 Mbp. The dashed lines of the same colour indicate similar patterns of variant distribution

The distribution of variants along the chromosomes revealed large regions with similar patterns of 10 kb peaks among the accessions (Fig. 3 and Supplementary Data S7), which could be a footprint of common ancestral introgressions or introgression of genetic material from one accession into another. SP accessions presented most of those regions, being SP1 the highest and followed by the other two Peruvian SP accessions, SP3 with 26 regions and SP2 with 22, and finally SP4 with 13. Regarding the SLC set, SLC3 displayed the highest number of regions sharing the same pattern of variant distribution with 12, followed by SLC1 with 11, SLC4 with five and finally SLC2 with only one region. Most of those regions were found in homozygous variant distribution compared to the heterozygous one, as the peak signals of the latter were generally weaker, which difficulted their identification. Nevertheless, many other regions shared highly similar variant distribution patterns, some of them in multiple accessions. The three SP from Peru shared the highest number of common variant distribution regions, being SP1 and SP3 the ones with the highest number (12), followed by SP3 and SP2 (eight), SP1 and SP2 (seven), SP4 and SLC1(both from Ecuador; four) and three shared variant distribution regions for SP2 and SP4, SP1 and SLC3 and SP3 and SLC3; other pairs of accessions presented two or less.

**Fig. 4:**
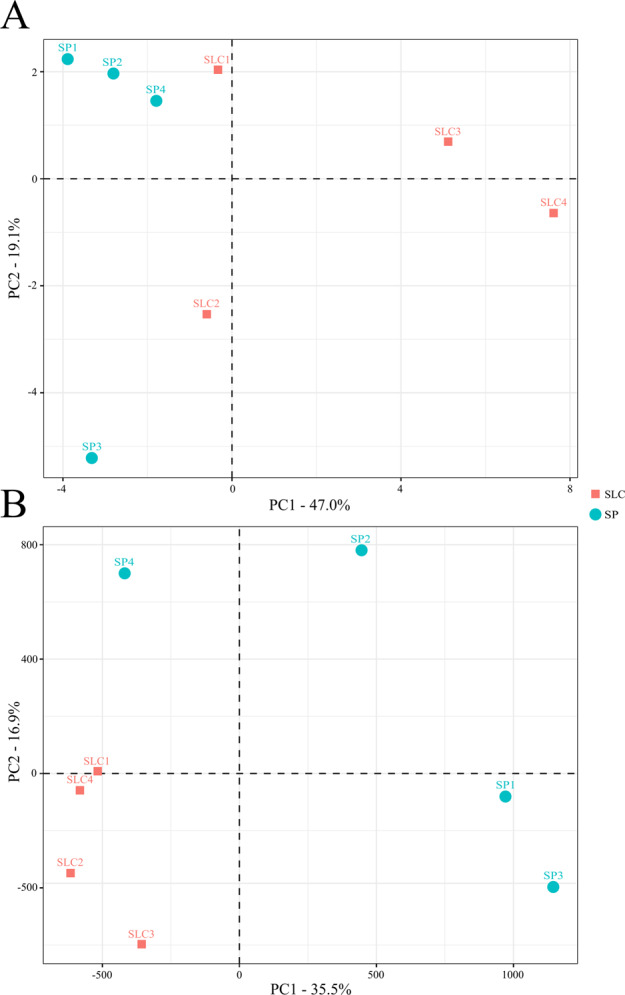
Principal component analysis (PCA) comparison. PCA similarities based on the morphological characterization of 39 traits (**a**) and on the whole set of SNPs identified in this study (9,106,964) (**b**) for the four *S. pimpinellifolium* (SP1–SP4) and four *S. lycopersicum* var. *cerasiforme* (SLC1–SLC4) accessions. The first and second principal coordinates (PC) are displayed. Study codes as in Table 1

The PCA made with the whole set of SNPs reflected the genetic relationships among the accessions and between the SP and SLC groups (Fig. 4b). The PC1 and PC2 accounted, respectively, for 35.5% and 16.9% of the genetic variation. The closer distribution of the SLC accessions in the PCA scatterplot in comparison with the wider distribution of the SP ones clearly demonstrates the narrowing of the genetic basis of SCL compared to the one of SP. It is also evident the greater proximity of SP4 to SLC, and particularly to SLC1, an intermediate accession between the two species, both coming from north Ecuador. Regarding the SP accessions, it is also evident their remarkable genetic differentiation following a clinal distribution from the south of Peru, (SP3), to the north of Ecuador (SP4), in agreement with what was found by Zuriaga et al.^10^. This means that a great part of the genetic diversity of this species has been captured in the four SP accessions selected as parents of our MAGIC population.

### Variants and gene annotation

The vast majority of the variants, more than 98% on average, were predicted by SnpEff as “modifier” (i.e. usually non-coding variants or variants affecting non-coding genes) suggesting that the prediction of their impact factor is difficult to estimate or there is no evidence of impact (Supplementary Data S8). In SP, those effects were assigned to slightly more than 7 million variants and ranged from 5.5 in SP4 to 7.8 million in SP1, versus 2.6 million in SLC, with a range from 2.2 in SLC3 to 3.3 million in SLC1. The second most abundant impact effects predicted were “moderate” (0.67% on average), from variants that might change protein effectiveness, being 49.1 thousand in SP, ranged from 39.9 in SP4 to 55.0 thousand in SP1, and 17.7 thousand in SLC, ranged from 15.3 in SLC3 to 20.7 thousand in SLC1. Subsequently, “low” impact effects (0.57% on average), which are mostly harmless variant or unlikely to change protein behaviour, were assigned to 44.2 thousand variants in SP, ranged from 34.8 in SP4 to 50.4 thousand in SP1, and 13.9 thousand in SLC, ranged from 12.0 in SLC3 to 17.2 thousand in SLC1. Finally, “high” impact effects (0.10% on average), corresponded to those variants that may have a disruptive impact on proteins like truncation or loss of function, were predicted in 6.2 thousand variants in SP, ranged from 5.4 in SP4 to 50.4 thousand in SP3, and 3.2 thousand in SLC, ranged from 2.9 in SLC3 to 3.4 thousand in SLC1. Of the “high” impact effects category, where most of the effects were found in “exon” and “splice sites”, the most abundant effect was “frameshift variant” (0.063%) caused by an insertion or deletion, followed by “stop gained” (0.020%) leading to a shorter transcript due to a premature stop codon, “splice acceptor variant” and “splice donor variant” (0.009%) when a variant hits two bases before the exon start or after the exon stop, respectively, “stop lost” (0.006%) leading to a longer transcript due to the loss of the stop codon, and finally “start lost” (0.005%) when the variant causes the substitution of a functional start codon to a non-functional (Supplementary Data S8).

Regarding the effects on protein function, on average, 42.5% of the variants were predicted to produce a silent effect, 56.1% a missense impact and 1.3% a nonsense protein product. Specifically, in SP an average of 898 variants were estimated to produce a nonsense impact, ranging from 826 in SP2 to 974 in SP3, and 479 in SLC, ranged from 451 in SLC1 and SLC2 to 531 in SLC4. Amino acids replacements and codon changes are reported in detail in Supplementary Data S9.

The GO term enrichment analysis of the variants with “high” impact effects revealed the genes, functions and traits that might have been mutated during speciation and domestication. Regarding the biological processes (BP) GO terms, significant common terms were found among all the accessions like “transmembrane transport” (GO:0055085), “RNA methylation” (GO:0001510), “mature ribosome assembly” (GO:0042256), “7-methylguanosine RNA capping” (GO:0009452) and “lipid glycosylation” (GO:0030259) (Supplementary Data S10). On the contrary, other BP GO terms were found only in SP accessions, like “beta-glucan biosynthetic process” (GO:0051274, SP1 and SP4), “oxidation-reduction process” (GO:0055114, SP1 and SP4) and “cell-wall modification” (GO:0042545, SP1, SP2 and SP4). Nevertheless, significant specific BP GO terms were found for each accession, some of them of great interest as all, or almost all, of the annotated terms were significant. For example, the term “GO:0006269” in SP2 (DNA replication, synthesis of RNA primer), associated with genes Solyc04g045530 and Solyc08g082200, where two out of two annotated terms were significant or “GO:0000290” in SLC4 (deadenylation-dependent decapping of nuclear-transcribed mRNA), associated with genes Solyc01g009390 and Solyc09g010300 where two out of three annotated terms were significant. The significant cellular component GO terms were related mainly with “membrane” (GO:0016020 and GO:0016021) and “chloroplast” (GO:0009507), along with significant accession-specific terms as in SP3 (Supplementary Data S11). Finally, for molecular function (MF) GO terms, both shared and accession-specific terms were found enriched for “high” impact effect variant, some of them showing all the annotated term significant (Supplementary Data S12). For example, in SP1, SP3 and SLC2 the term “GO:0010309” (acireductone dioxygenase [iron(II)-requiring] activity), was found significant for all the three genes in which it was annotated (Solyc09g082630, Solyc09g082640 and Solyc09g082650) or in SP2 the term “GO:0003896” (DNA primase activity) found significant for the two genes annotated (Solyc04g045530 and Solyc08g082200). The list of genes associated with significant GO terms is reported in Supplementary Data S13.

Sixteen genes described in the literature as candidates to control some of the morphological traits evaluated in this study plus biotic resistances were evaluated for impact variants predicted by SnpEff (Supplementary Data S14). Impact variants were found in all the genes analysed except for the genes *I2* (Solyc11g071430; *Fusarium oxysporum* f. sp. *lycopersici* resistance), *Ph3.2* (Solyc09g092310; *Phytophthora infestans* resistance) and *Pto* (Solyc05g013300; *Pseudomonas syringae* pv. *tomato* resistance). Genes *ty-5* (Solyc04g009810; tomato yellow leaf curl virus resistance), *Ve2* (Solyc09g005080; *Verticillium albo-atrum* resistance), *Ph3* (Solyc09g092280; *Phytophthora infestans* resistance) and *Me* (Solyc02g081120; leaf complexity), *style2.1* (Solyc02g087860, stigma exertion) presented only “moderate” or “low” variants. The gene that presented a higher number of “high” impact variant was *cf2* (solyc06g008300; *Cladosporium fulvum* resistance) with four variants, followed by *Ve1* (Solyc09g005090; *Verticillium albo-atrum* resistance) and *bsr4* (Solyc05g007850; *Xanthomonas campestri*s pv. *vesicatoria* resistance) with three variants, and *Pfr* (Solyc05g013280; *Pseudomonas syringae* pv. *tomato* resistance) with two variants. The remaining genes, *coi1* (Solyc05g052620; *Pseudomonas syringae* resistance), *Lin5* (Solyc09g010080; invertase), *asc* (Solyc03g114600; *Alternaria alternata* f. sp. *lycopersici* resistance), *lyr* (Solyc05g009380; reduced leaf complexity), *Sw-5* (Solyc09g098130; tomato spotted wilt virus) carried one “high” impact variant. Regarding the accessions, SP2 and SP4 showed the highest number of “high” impact variant with seven in five and six genes, respectively, followed by SLC1 and SLC4 with six in six and five genes, SP3 and SLC2 with five in four and five genes, SP1 with four in four genes and finally SLC3 with three in three genes.

## Discussion

We performed a comprehensive phenotypic characterization and whole-genome resequencing of eight accessions representing the range of diversity of the closest relatives of domesticated tomato. These materials, which originated from different geographic regions of south and central America, were selected to maximize important morphoagronomic traits and genetic diversity. In order to harness this valuable diversity, these accessions have been used as founders of a MAGIC population that is currently under development^39^. MAGIC populations have demonstrated extraordinary precision to detect candidate QTLs and traits interactions, as well as markers and traits association with greater efficiency than other experimental populations^40,41^. Therefore, the information provided in this study will greatly improve recombination detection, haplotype prediction and causal variant identification in the MAGIC population. In fact, even though hundreds of tomato and wild relatives accessions have been already re-sequenced^7,42–44^, only the sequencing of the MAGIC founders can efficiently exploit the whole potential of MAGIC populations^45–47^.

On the other hand, even though a MAGIC intraspecific population is already available^45^, to our knowledge, no interspecific MAGIC population has been developed using such a substantial proportion of the fully cross-compatible wild and weedy tomato genetic and morphological diversity. It is well known that most of the cultivated species, including tomato, have a narrow genetic diversity due to domestication and breeding processes^48^. Nevertheless, new commercial varieties may show higher phenotypic and especially genetic diversity compared to heirlooms due to the recent introgressions of regions/alleles from wild species, like abiotic and biotic resistance or rescuing alleles for flavour improvement^5,7,49^. In fact, in the last decade, there is a resurgence of introgression breeding, thanks to international campaigns, like “The CWR Project” (https://www.cwrdiversity.org/project/), and new breeding approaches, such as “Introgressiomics“^50^, driven by the new global challenges like climate change^51^. Thanks to the precision provided by the MAGIC populations for genomic studies coupled with the resequencing of its founders, we aim at identifying candidate QTLs and causal variants for incorporation into breeding pipelines for developing new resilient and enhanced quality tomato varieties, as well as to shed light on the genetics of domestication traits.

### Morphological variation

The eight accessions selected exhibited a great morphological diversity with regards to plant, inflorescence and fruit traits. Regarding SP, a remarkable morphological variation was already observed by Rick et al.^17,52^. These authors found a high correlation between variation in flower size and stigma exertion with cross-pollination, giving rise to a higher rate of crosses among plants of different populations and contributing to the increase of variation. This morphological variation was revealed also in a more recent study conducted by Blanca et al.^4^. In this study 63 accessions, covering the distribution range of this species were genetically and morphologically characterized. Morphological variation was found for all plant organs, associated to the geographical origin. Thus, hairless plants with long trusses and many flowers per truss, flowers with big petals and exerted stigmas and small, round and intense red fruits, were more common in the North of Peru, considered the centre of origin of this species. As plants moved away from this area, they displayed changes in their morphology, presumably because of the adaptation to different climatic conditions^53^. The four accessions of SP studied in our work show clear morphological variations that fit with this evidence. SP1 was collected in Piura, located in the tomato centre of origin, and exhibits the typical characteristics detailed before. The most morphologically different accessions were SP3, and SP4. SP3 was collected in Ica, a very dry area in the coastal part of Peru, and SP4 comes from Manabí, a hot and wet area in Ecuador. SP3 showed an intense anthocyanin pigmentation on the stem, a characteristic that clearly shows its adaptation to abiotic stresses such as water deficit^54^, while SP4 exhibited bigger fruits and leaves, more adapted to humid conditions. In both cases, the stigmas were inserted suggesting a transition from a predominantly allogamous reproduction in the centre of origin, to a predominantly autogamous one, a clear change when species migrate from its centre of origin to distant areas^55^. SP2 collected in the Amazonas province was the most similar to SP1 and both accessions were also the closest geographically.

Regarding SLC, the variation observed was also noticeable. SLC1 grouped in the PCA with three of the SP accessions, and more closely to SP4. SLC1 exhibited intermediate morphological characteristics between SP and SLC. In fact, the range of morphological variation between SP and SLC is continuous, existing intermediate forms as a result of naturally occurred crosses in areas where both species are sympatric. These intermediate forms were also detected by Blanca et al.^4^. The other accession that groups near the SP accessions in the PCA is SLC2. This accession was collected as a wild specimen in the Sinaloa desert in Mexico. In this country, SLC is widely distributed, and it can be found in tropical and subtropical areas with semiarid or humid regimes as a semi-wild or weed, in this latter case on many occasions being tolerated in cultivated biodiverse orchards^56^. Wild accessions coming from desert areas have usually smaller fruits, resembling those of SP, explaining its grouping with SP accessions in the morphological PCA. Finally, SLC3 and SLC4 are the most similar to the cultivated tomato in terms of morphological traits, and consequently, they group far from the other accessions in the PCA. In fact, the existence of high variability in morphological traits in SLC was already highlighted by Rick and Holle^57^ and corroborated later by Blanca et al.^4^ and Mata-Nicolás et al.^58^, who demonstrated the presence of *fas* (fasciated), *loc* (LOC), and *fw2.2* and *fw3.2* (FW) and *ovate* alleles in SLC coming from Ecuador and Peru, which was determinant to the increase of FW and diversification of FS. In some cases, SLC has been even sold in local markets^59^ and, for this purpose, some unconscious selections may have been performed by growers, mainly increasing its fruit size. As shown in the PCA, the wide distribution of SLC accessions demonstrates that these accessions hold a considerable amount of the morphological variation found in this species.

#### Mapping, variants and phylogenetic relationships

For this study, the latest version of the reference genome Heinz 1706 (version SL4.0)^26^ was used to map the high-quality reads of SP and SLC accessions. A comparison with the previous version of the reference genome (version SL3.0, data not shown) revealed a substantial improvement in mapping rates, variants identification and other statistics, reflecting the assembly quantum leap of the latest version. The lower mapping rates of SP compared to SLC accessions is certainly due to the higher phylogenetic distance of the former^4^. The average of 5% of SP and 3% of SLC reads that did not map to the reference genome may host genomic regions that have been lost due to domestication and breeding processes^42^. The pan-genome analysis of 725 accessions suggested the loss of ~200 genes within SP in northern Ecuador, followed by a continuous process of additional gene losses during the SLC pre-domestication in Mesoamerica and the SLL domestication in Mexico^7^. Likewise, tomato improvement contributed to gene loss, although at a lesser extent than domestication. Analysis of allelic frequencies revealed that 120 and 1,213 genes showed, respectively, higher and lower frequencies in SLC than in SP, which would be a consequence of the domestication process, while only 12 and 665 genes had higher and lower frequencies, respectively, in SLL heirlooms than in SLC, which would be a result of the breeding process, pointing to a major gene loss during domestication than in the breeding process^7^. Resequencing and new genome assemblies of SP and SLC accessions will enable the identification of loss genes and genomic regions of tomato relatives that may hide interesting traits for tomato improvement.

A total of 12,833,972 variants were identified among the MAGIC founders, a 2.9-fold more than those identified in the founders of the tomato intraspecific MAGIC^60^. Higher number of available variations may be translated in an ultra-dense genetic map for MAGIC that will reduce QTL intervals and increase the detection of candidate genes and causal variants. In this study, we maximized the genetic diversity of the founders within SP and SLC groups, selecting those accessions that showed the highest diversity in a large panel of samples assessed with a robust set of SNPs^5^. The set of variants identified in this study, which is over 1,600-fold more abundant the one used by Blanca et al.^5^, confirmed those results, with higher precision and avoiding the bias due to the SNP selection of a genotyping array. SP accessions showed higher genetic diversity than SLC as confirmed by several studies^5,7,43^. SP accessions from Peru (SP1, SP2 and SP3) displayed more variants than the SP4 from Ecuador, yet the latter presented a high diversity, as well a high number of private variants, compared to the reference genome. On the opposite, SLC1 from Ecuador showed more variants than SLC3 from Peru, which agrees with the morphological data, which revealed its intermediate morphology between the extremes of variation of both taxa. As expected, the SLC2 from Mexico, which is considered the region where SLL was domesticated from SLC showed less diversity with respect to the tomato reference genome than any other SLC accession. On the contrary, surprisingly, SLC4 from Costa Rica showed several polymorphisms between SLC1 and SLC3, which confirms the complex evolutionary history of SLC from the Andes to Mesoamerica^6^.

Those results are also reflected in the genetic PCA (Fig. 3b), where the distribution of the samples is alike the arch-like structure from south Peru to North Ecuador of Blanca et al.^5^. The base of the arch started with SP3, from Ica (Peru) the accession with the highest number of polymorphisms, followed by SP1 and SP2 (from Piura and Amazonas provinces of Peru, respectively), the second and third accessions with higher variants number, all from Peru. The second half of the arch, which is clearly defined by the PC1, started with SP4 and followed by SLC1, both from Ecuador, then SLC4 from Costa Rica and finally SLC2 from Mexico and SLC3 from Peru. The stepwise SLC flow from Ecuador to Central America, like Costa Rica, to Mexico, supporting the hypothesis of the two-step domestication process that has been suggested in previous studies^5,43^, although it does not rule out a more complex hypothesis^6^. Regarding the position of SLC3 from Peru as the left base of the PCA arch, Blanca et al.^5^ also suggested a second domestication hypothesis where SLC could have reached Mesoamerica in one step from Northern Peru based on the allele frequency of FW and FS genes. In any case, both hypotheses suggested that the SLC migration to Mexico resulted in a strong genetic bottleneck^5,43^, which is also reported in our study where the SLC accessions clustered in one PCA quadrant.

Another way to detect genetic similarities and reconstruct domestication processes is by investigating the variant distribution pattern along the chromosomes as a footprint of common history or potential introgressions^42^. The three SP accessions from Peru presented most of the identical variant distribution regions, sharing significantly less with SP4 from Ecuador. The latter showed the highest number of potential introgression (four) between one SP and one SLC (SLC1), both from Ecuador, followed by SP1 and SLC3 and SP3 and SLC3, all from Peru. SLC3 and SLC1 showed a relatively high number of those regions, an evidence of the complex domestication history of SLC, especially in Peru and Ecuador^5,6^. Contrastingly, SLC4 from Costa Rica and SLC2 from Mexico shared only five and one variant distribution regions, respectively, suggesting strong selective pressure of SLC during its migration to Mexico. With a considerably larger panel of accessions, representing all the genetic and geographic subpopulations of tomato relatives, this approach could be helpful in shedding light on phylogenetic relationships of the two taxa and domestication history for a more efficient introgression breeding.

#### Functional annotation

The Gene Ontologies analyses associated with genes with “high” impact variants could reflect some patterns related to domestication processes among SP, SLC and SLL, the latter represented by the reference genome of variety Heinz 1706. For example, all the SP accessions showed the BP GO parent term “cell-wall modification” (GO:0042545) as one of the most significant, which child terms are “plant-type cell-wall modification” (GO:0009827), “cell-wall thickening” (GO:0052386) and “cell-wall modification involved in multidimensional cell growth” (GO:0042547). This parent term is associated with dozens of genes, mostly related to the pectinesterase activity, as “Solyc01g067410”, “Solyc01g067420” or “Solyc01g079180”, among others. Pectinesterase is a ubiquitous cell-wall-associated enzyme that catalyses the demethylation of pectin and it is involved in many developmental processes like stem elongation^61^, pollen tube development^62^, abscission^63^, pathogens and herbivore attack^64^ and fruit ripening^65^. In tomato, the silencing of *Pmeu1*, a ubiquitously expressed pectinesterase gene, resulted in enhancement of the rate of softening during ripening^66^. Likewise, pectinesterase genes are thought to increase fruit susceptibility to blossom‐end rot increasing Ca^2+^ bound to the cell wall and decreasing Ca^2+^ available for other cellular functions^67^. The same GO Term “GO:0042545”, as well as others found in our study, like “GO:0055085”, “GO:0001510” or “GO:0042256”, has been associated with genes that were negatively selected during domestication and improvement^7^. Most of those genes were directly or indirectly related to defence response, like cell-wall thickening, which acts as a barrier against biotic and biotic stresses and contributes to fruit firmness and flavour.

The evaluation of the candidate genes found 43 high-impact variants in all of them. Future work will confirm whether these variants detected in the analysed genes are responsible for the phenotypic variations. Despite no high-impact variants were found in all genes, this does not exclude that some of the variants labelled as moderate are not responsible for important gene changes. Indeed, some of the variants responsible for high effect on the phenotype are located outside of the coding regions, or are due to inversions, such as the genes that control the FS^68^.

On the other hand, no variants were found in *Pto* (Solyc05g013300) gene although resistance against *race 1* of *Fusarium oxysporum* f. sp. *lycopersici* and *Pseudomonas syringae* pv. *tomato* race 0 has been detected among the eight accessions (Pereira-Dias et al., unpublished). This means that the resistance found could be due to resistance genes not described yet. In this way, the genetic analysis of the MAGIC population for tolerance to both diseases will be of great relevance to find candidate genes for this disease.

## Conclusions

In the present study, we performed an extensive phenotyping and a comprehensive structural and functional characterization through whole-genome resequencing of four *S. pimpinellifolium* and four SLC accessions. These eight accessions were selected to maximize the genetic and morphological diversity of both groups of tomato relatives to be the founders of the first interspecific MAGIC in tomato. The wealth of information provided in this study will help in gaining resolution in QTLs detection and candidate genes and causal variants identification for relevant morphological and agronomic traits. The future MAGIC lines that will carry a variable percentage of parent genome may be a genetic resource of interest to develop new resilient and high-quality tomato varieties.

## Supplementary information

Supplementary Data S1

Supplementary Data S1rev

Supplementary data S3

Supplementary data S4

Supplementary data S5

Supplementary data S6

Supplementary data S7

Supplementary data S8

Supplementary data S9

Supplementary data S10

Supplementary data S11

Supplementary data S12

Supplementary data S13

Supplementary data S14
