# Supplementary figures and images for: Morphoagronomic characterization and whole-genome resequencing of eight highly diverse wild and weedy S. pimpinellifolium and S. lycopersicum var. cerasiforme accessions used for the first interspecific tomato MAGIC population
Source: Hortic Res. 2020 Nov 1;7:174. doi: 10.1038/s41438-020-00395-w (PMC7603519; doi:10.1038/s41438-020-00395-w)

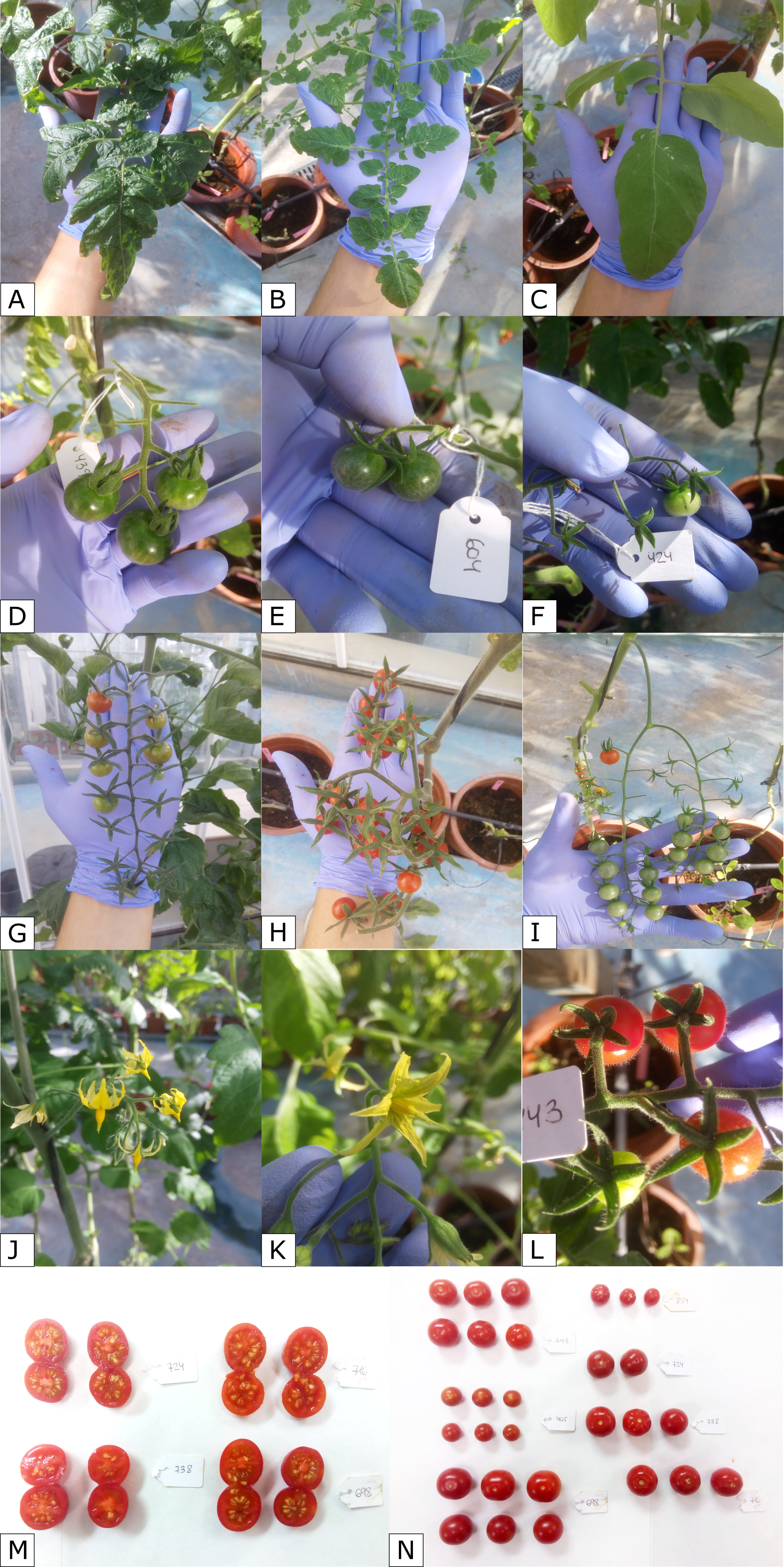

Supplement: Supplementary file 1 — Supplementary Data S1 [file 41438_2020_395_MOESM1_ESM.png]
