# Supplementary material for: Morphoagronomic characterization and whole-genome resequencing of eight highly diverse wild and weedy S. pimpinellifolium and S. lycopersicum var. cerasiforme accessions used for the first interspecific tomato MAGIC population
Source: Hortic Res. 2020 Nov 1;7:174. doi: 10.1038/s41438-020-00395-w (PMC7603519; doi:10.1038/s41438-020-00395-w)
Supplement: Supplementary file 7 — Supplementary data S7 [file 41438_2020_395_MOESM7_ESM.pdf]

Supplementary Data S7. Distribution of homozygous (red) and heterozygous (orange) variants along all the chromosomes for the four SP and four SLC accessions using Heinz 1706 SL4.0 as a reference genome[28]. The peaks represent high frequencies of variants in a window size of 10 Mbp. The dashed lines of the same color indicate similar patterns of variant distribution.

	SP1	SP2	SP3	SP4	SLC1	SLC2	SLC3	SLC4
**SP1**		7	12	2	1	0	3	1
**SP2**	7		8	3	2	0	1	1
**SP3**	12	8		2	1	0	3	1
**SP4**	2	3	2		4	0	1	1
**SLC1**	1	2	1	4		0	2	1
**SLC2**	0	0	0	0	0		0	0
**SLC3**	3	1	3	1	2	0		2
**SLC4**	1	1	1	1	1	1	2	
**Total**	26	22	27	13	11	1	12	5

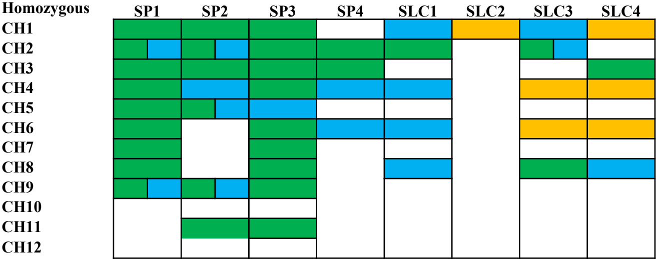

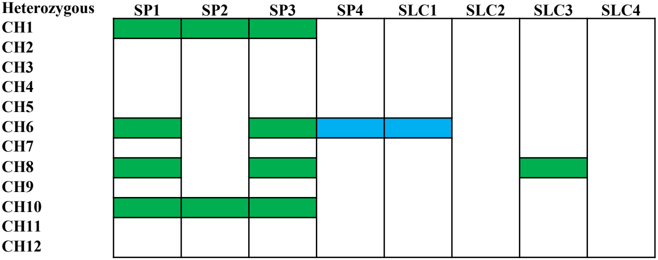

Chromosome 1 homozygous variants

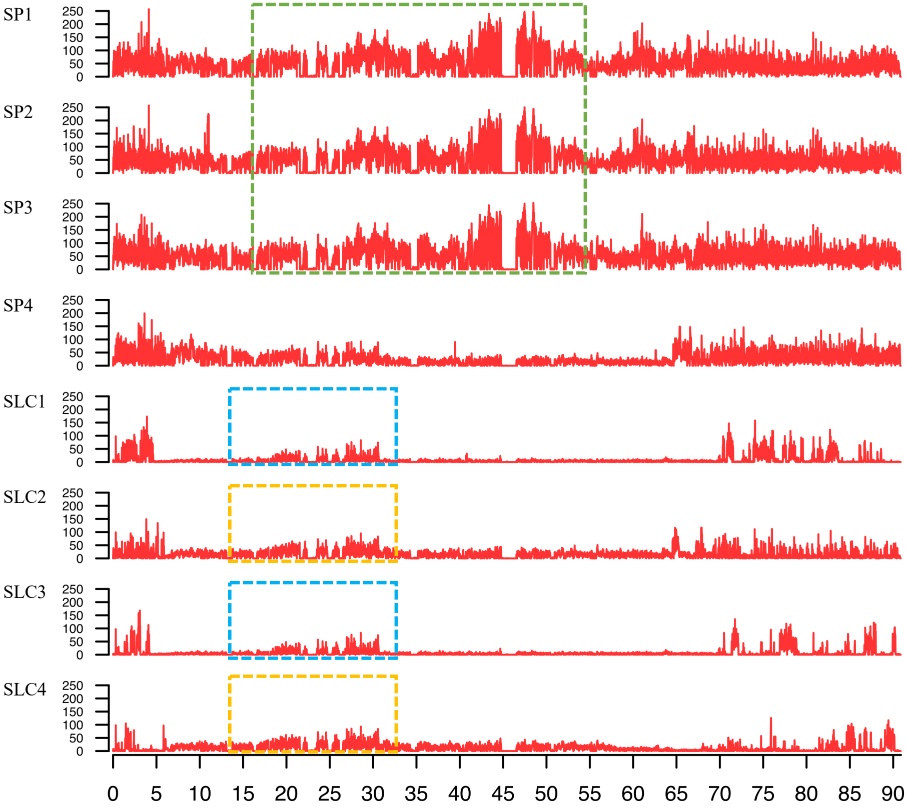

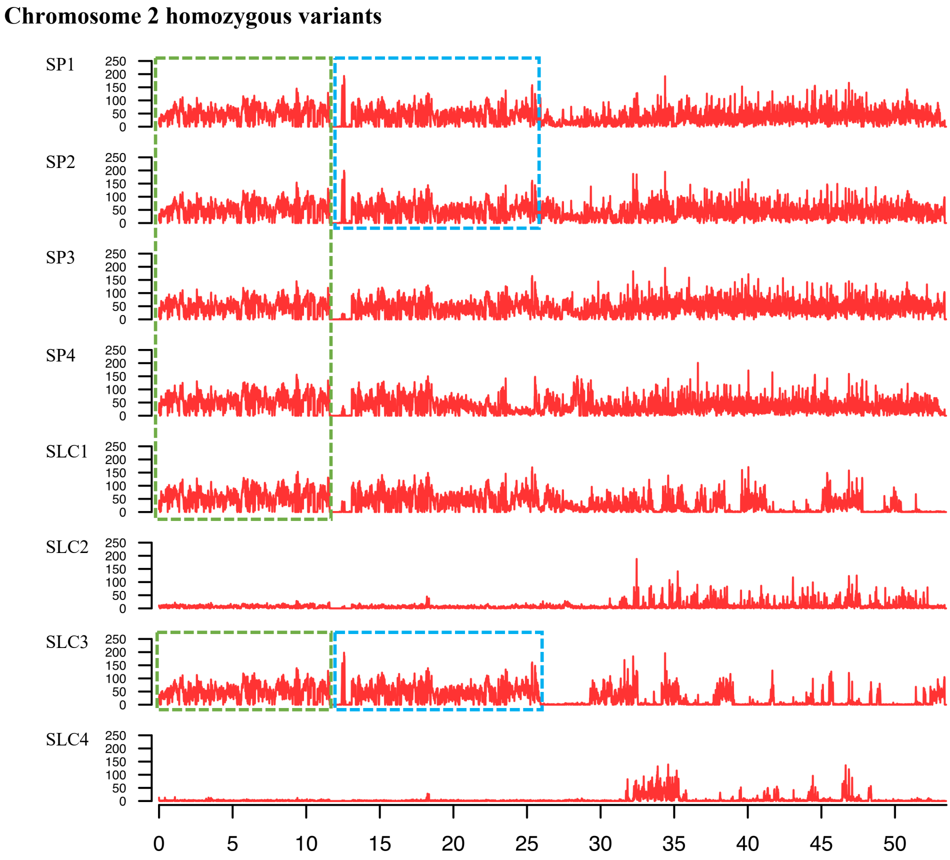

Chromosome 2 homozygous variants
SP1
SP2
SP3
SP4
SLC1
SLC2
SLC3
SLC4
250
200
150
100
50
0
0
5
10
15
20
25
30
35
40
45
50

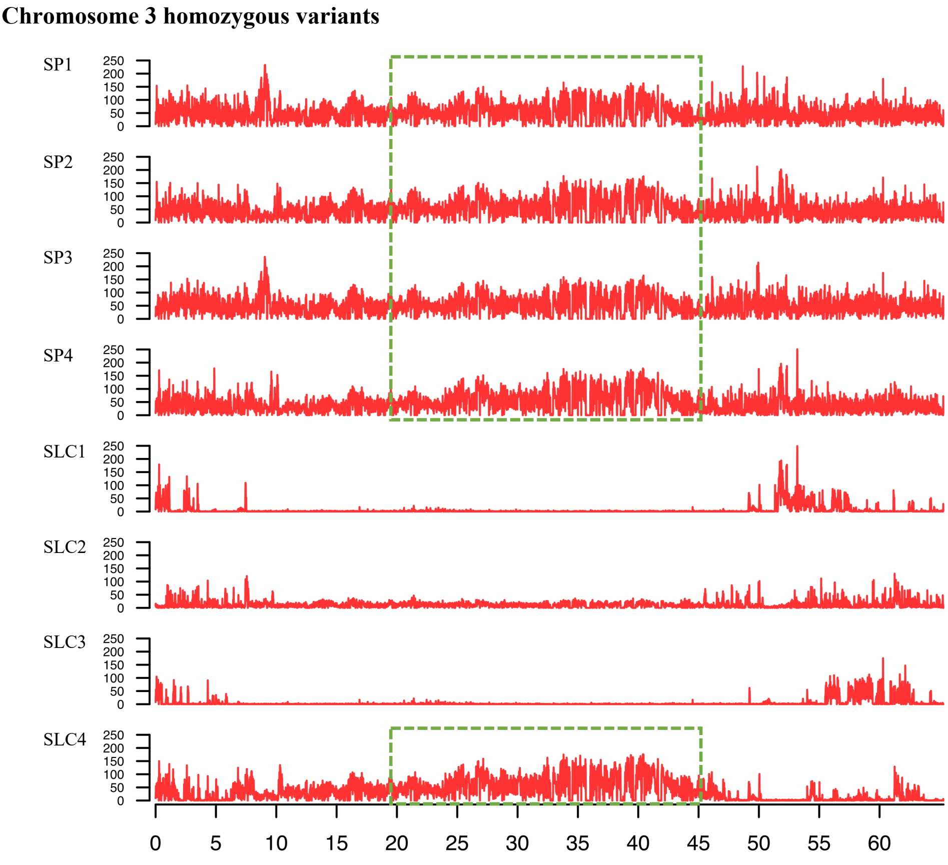
Chromosome 3 homozygous variants
SP1
SP2
SP3
SP4
SLC1
SLC2
SLC3
SLC4
250
200
150
100
50
0
0
5
10
15
20
25
30
35
40
45
50
55
60

Chromosome 4 homozygous variants

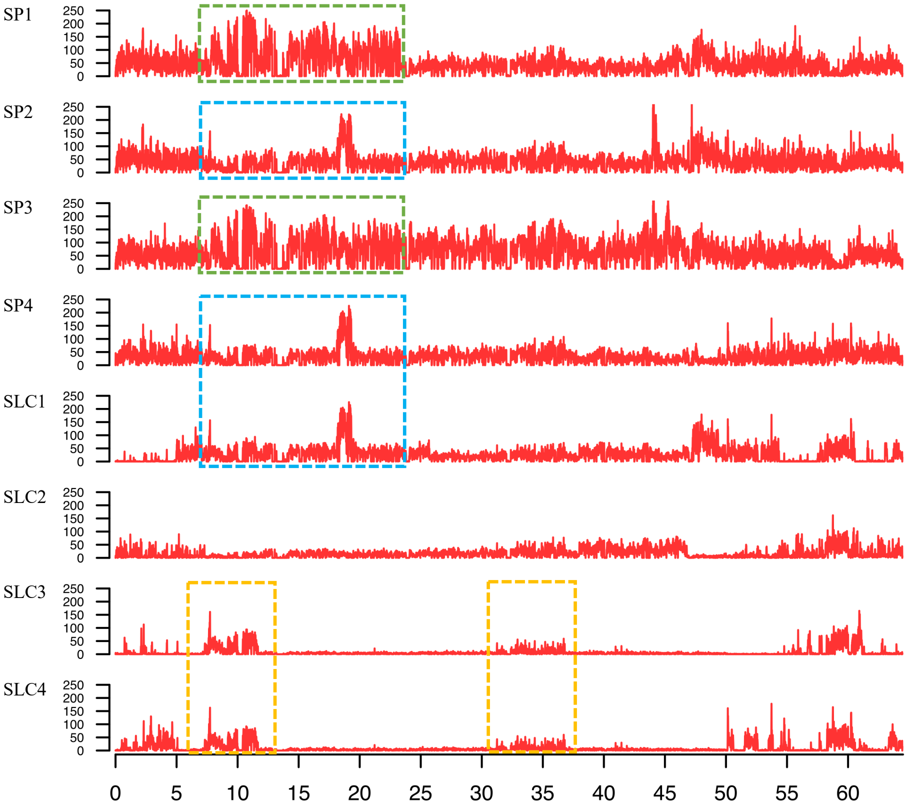

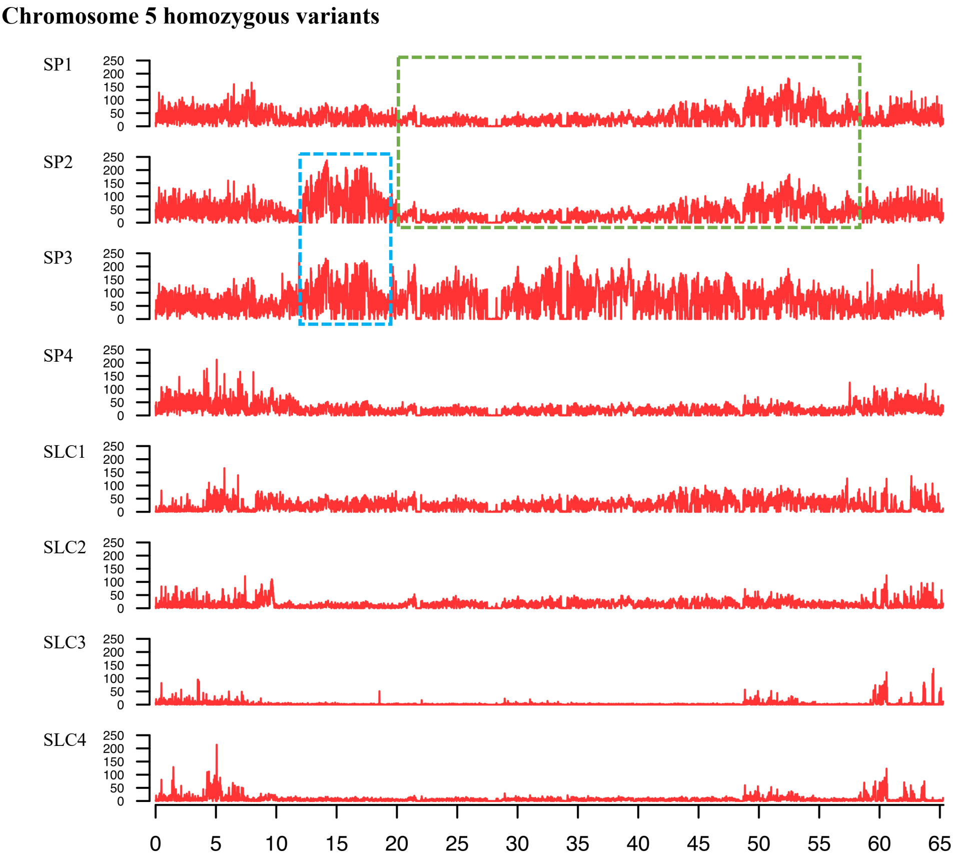

Chromosome 5 homozygous variants
SP1
SP2
SP3
SP4
SLC1
SLC2
SLC3
SLC4
250
200
150
100
50
0
0
5
10
15
20
25
30
35
40
45
50
55
60
65

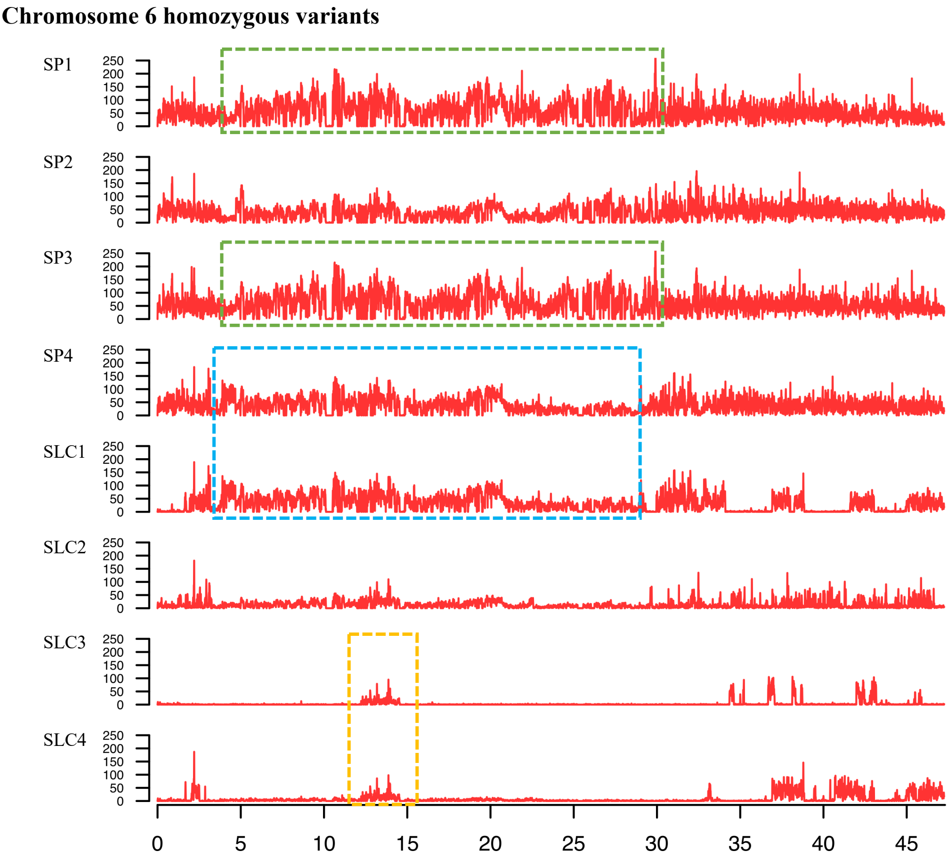
Chromosome 6 homozygous variants
SP1
SP2
SP3
SP4
SLC1
SLC2
SLC3
SLC4
250
200
150
100
50
0
0
5
10
15
20
25
30
35
40
45

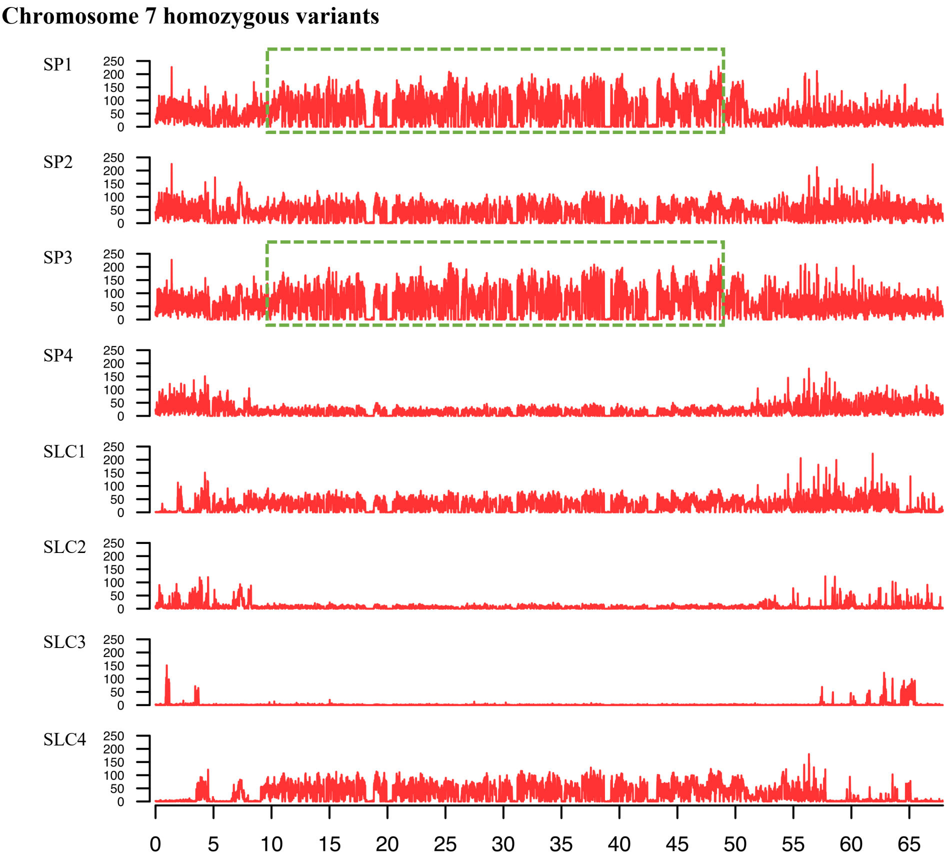

Chromosome 7 homozygous variants
SP1
SP2
SP3
SP4
SLC1
SLC2
SLC3
SLC4
250
200
150
100
50
0
0
5
10
15
20
25
30
35
40
45
50
55
60
65

Chromosome 8 homozygous variants

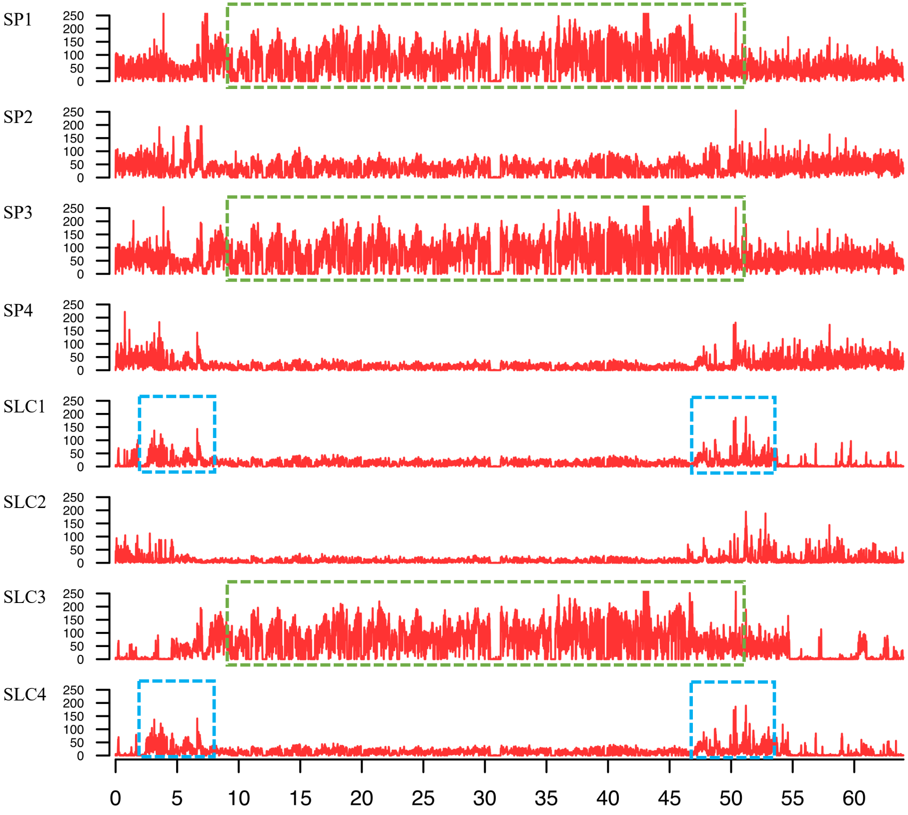

Chromosome 9 homozygous variants

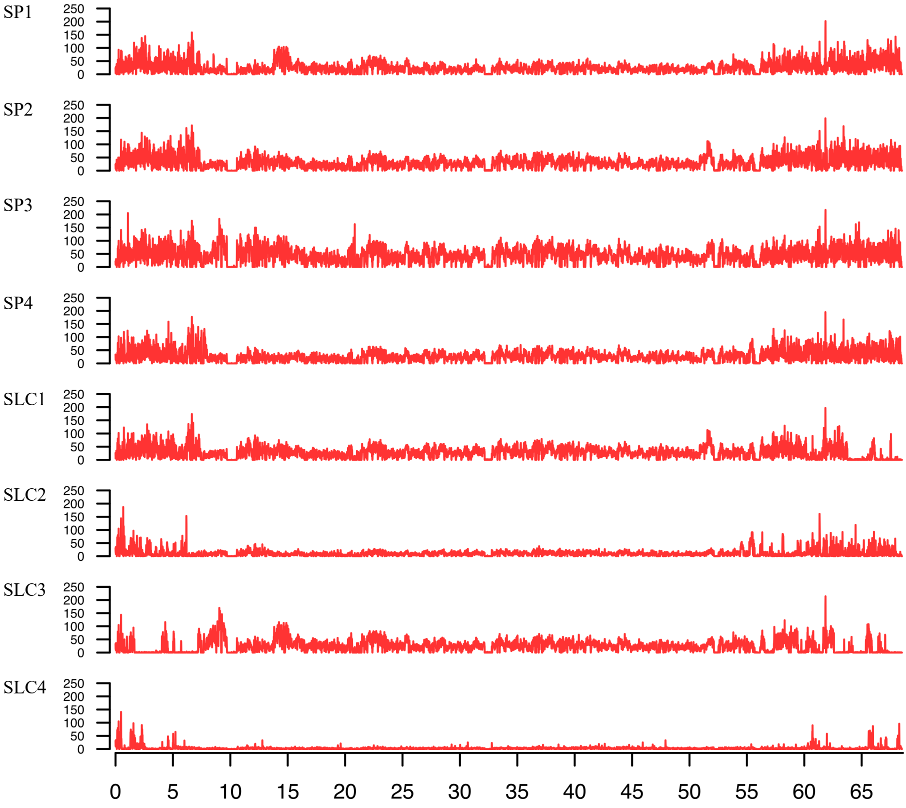

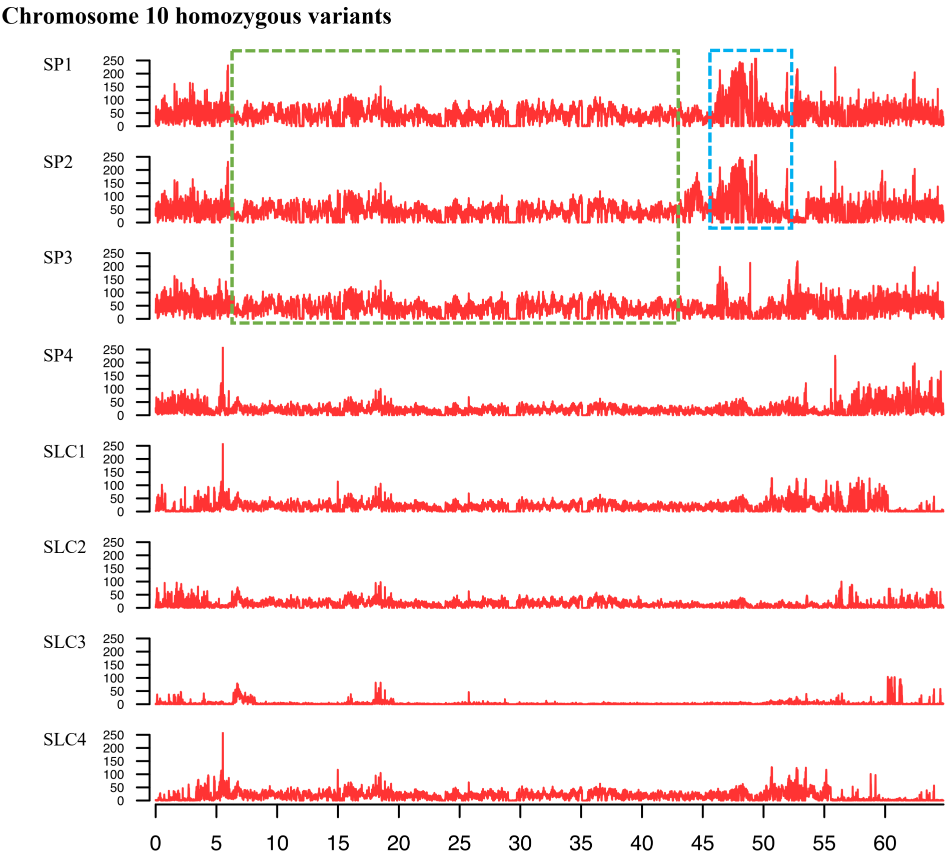

Chromosome 10 homozygous variants
SP1
SP2
SP3
SP4
SLC1
SLC2
SLC3
SLC4
250
200
150
100
50
0
0
5
10
15
20
25
30
35
40
45
50
55
60

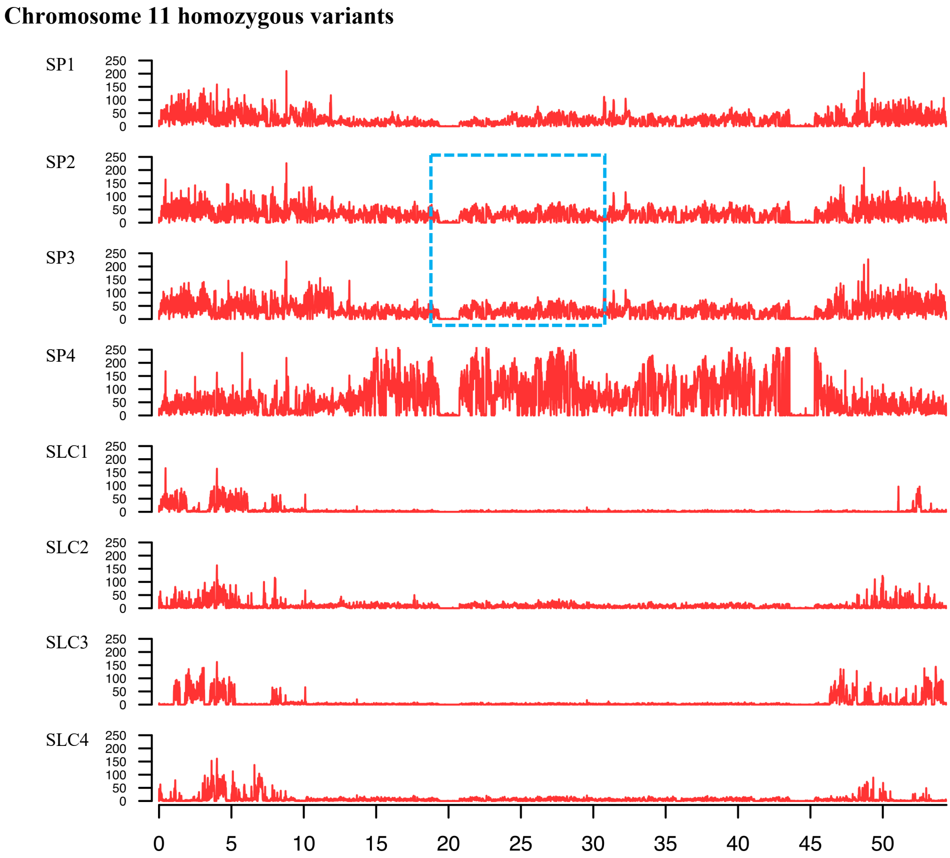

Chromosome 11 homozygous variants
SP1
SP2
SP3
SP4
SLC1
SLC2
SLC3
SLC4
250
200
150
100
50
0
0
5
10
15
20
25
30
35
40
45
50

Chromosome 12 homozygous variants

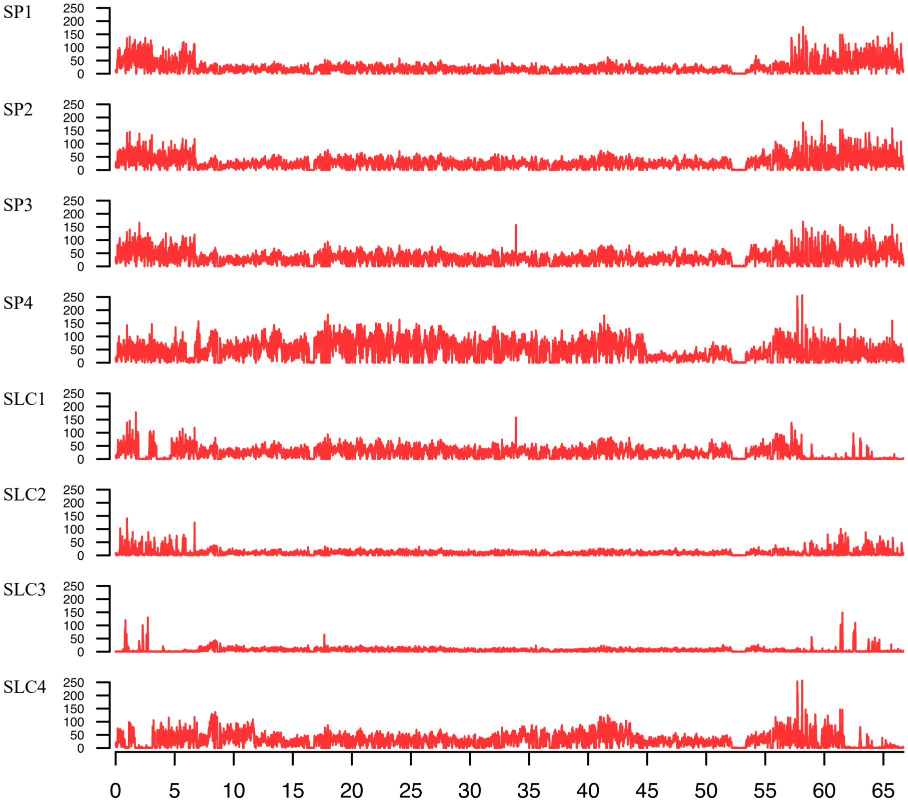

Chromosome 1 heterozygous variants

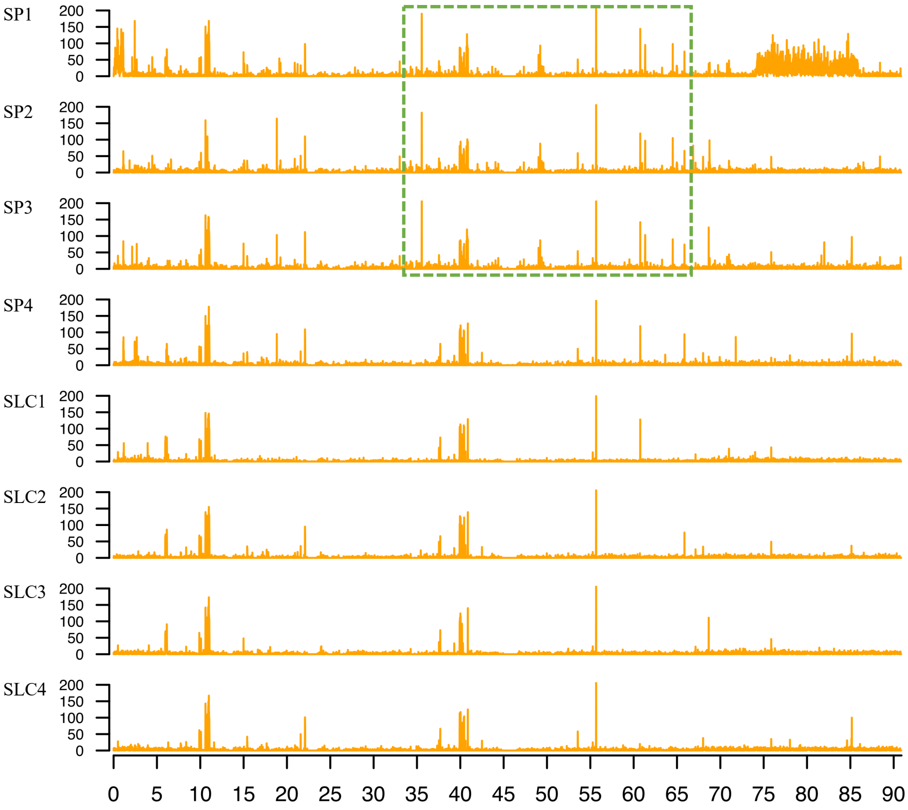

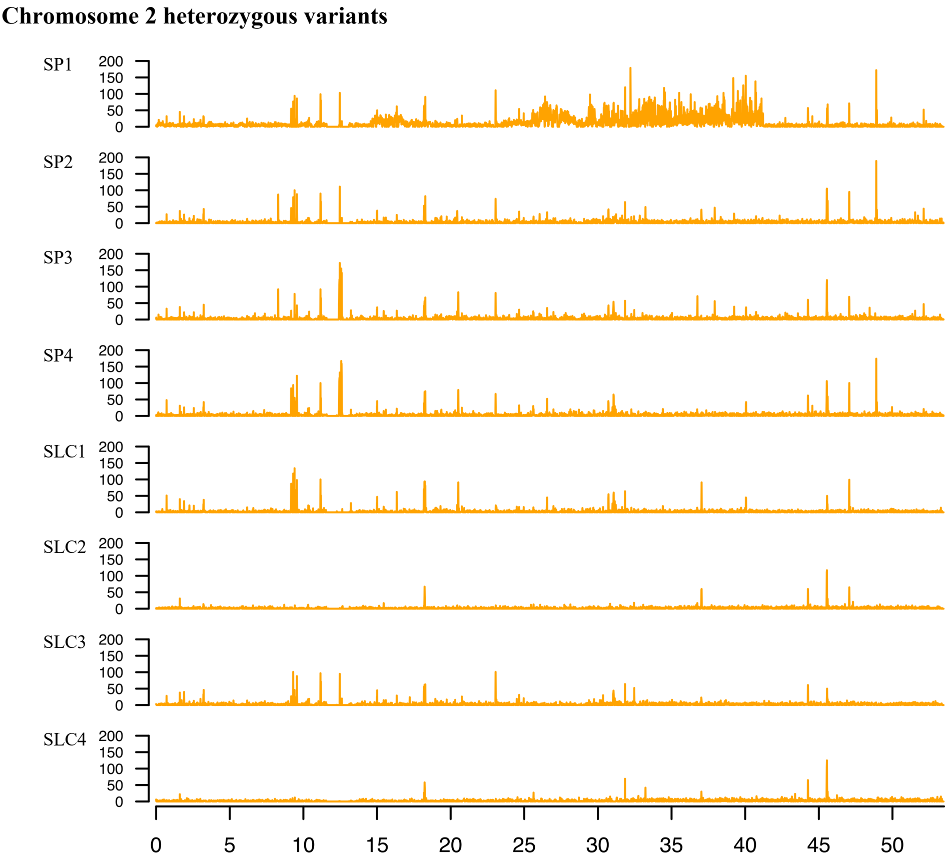

Chromosome 2 heterozygous variants
SP1
SP2
SP3
SP4
SLC1
SLC2
SLC3
SLC4
200
150
100
50
0
0
5
10
15
20
25
30
35
40
45
50

Chromosome 3 heterozygous variants

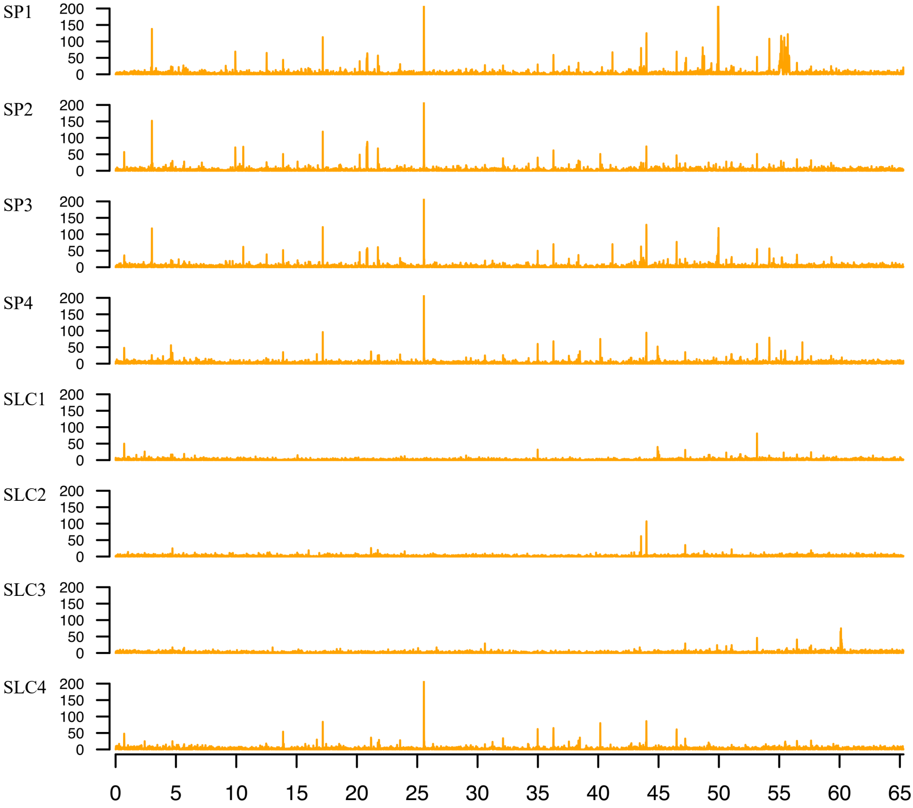

Chromosome 4 heterozygous variants

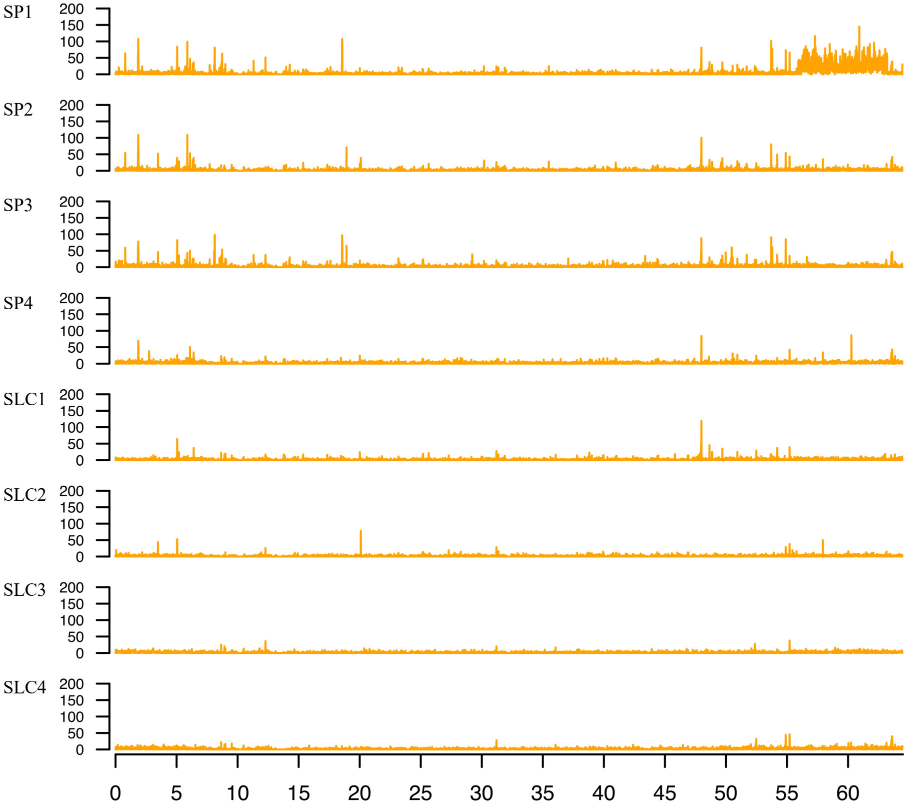

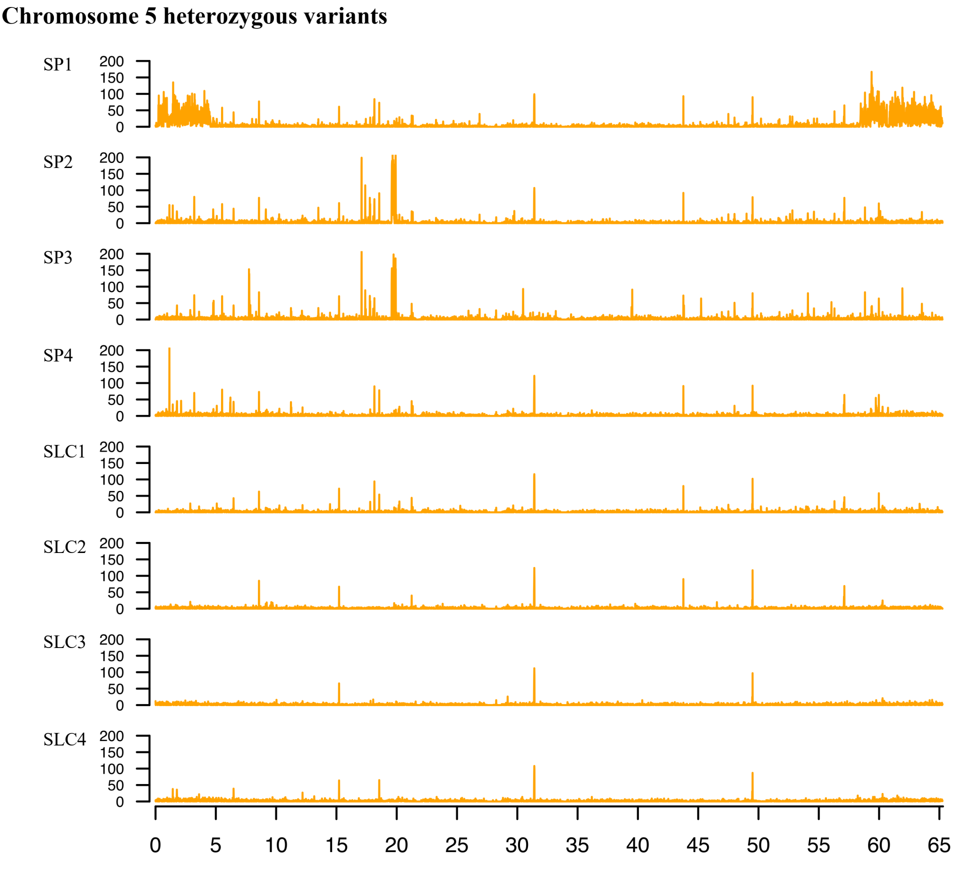
Chromosome 5 heterozygous variants
SP1
SP2
SP3
SP4
SLC1
SLC2
SLC3
SLC4
200
150
100
50
0
0
5
10
15
20
25
30
35
40
45
50
55
60
65

Chromosome 6 heterozygous variants

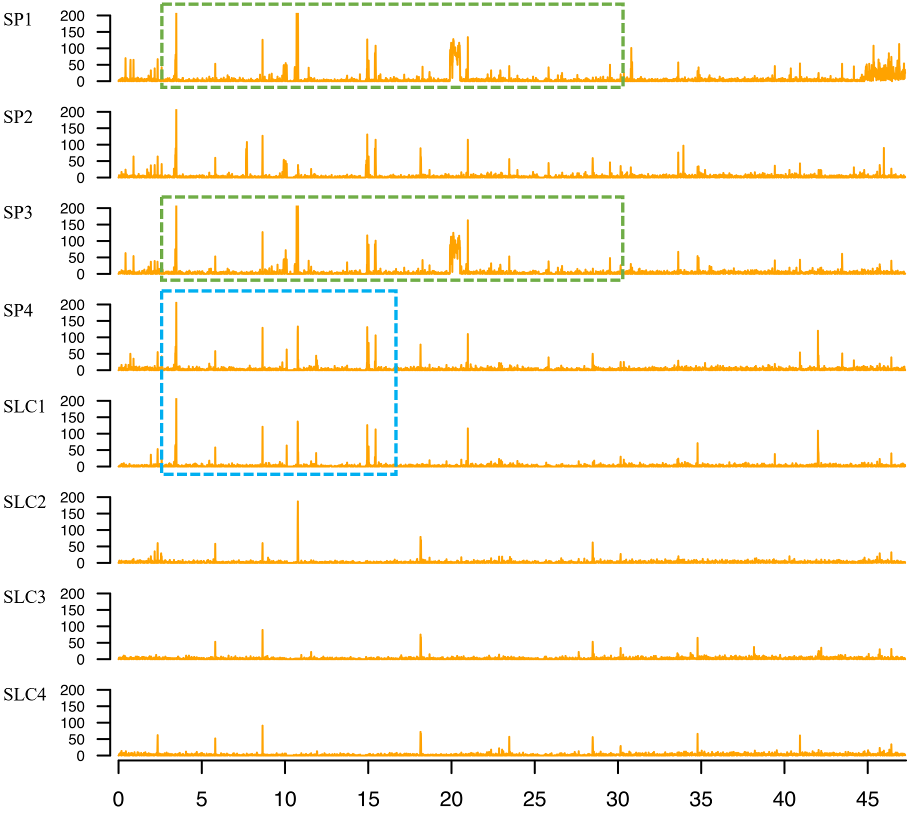

Chromosome 7 heterozygous variants

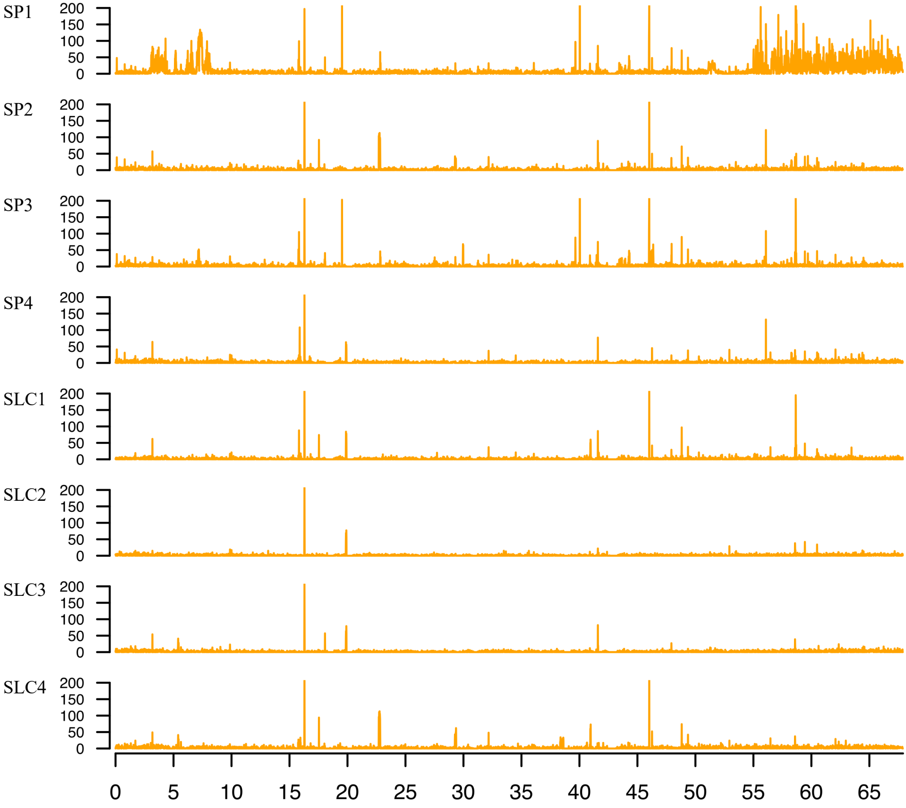

Chromosome 8 heterozygous variants

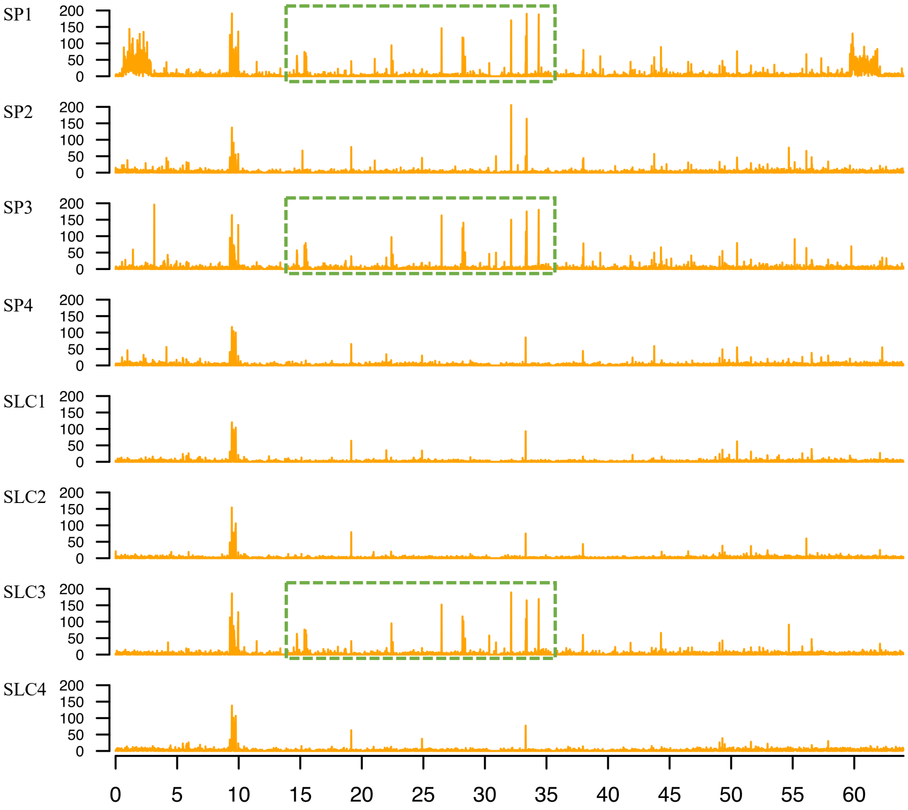

Chromosome 9 heterozygous variants

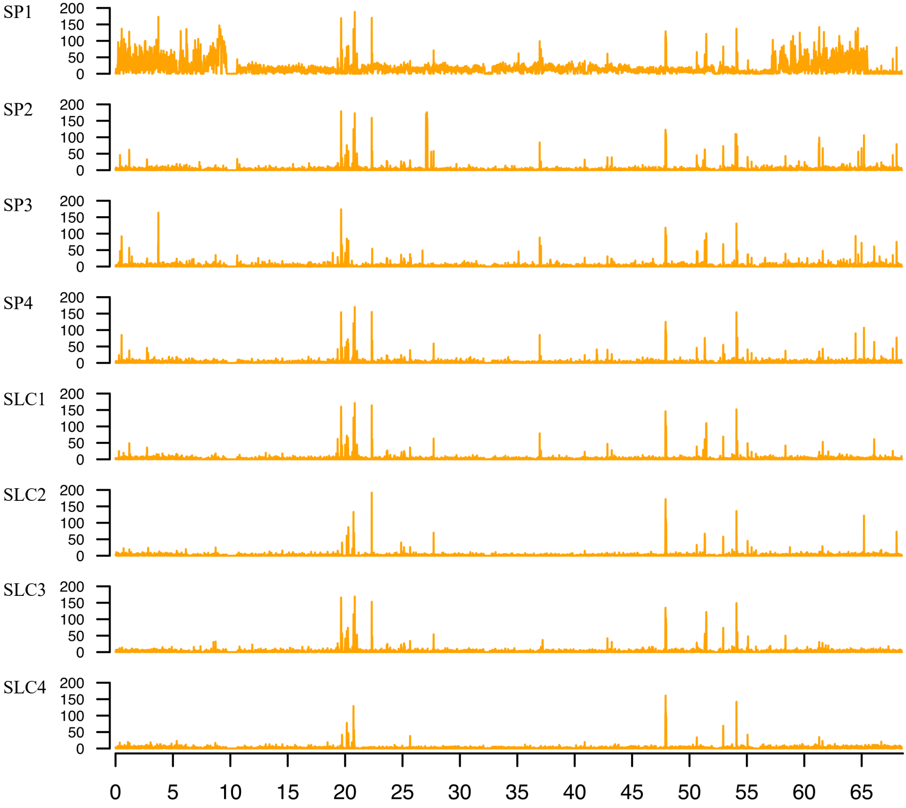

Chromosome 10 heterozygous variants

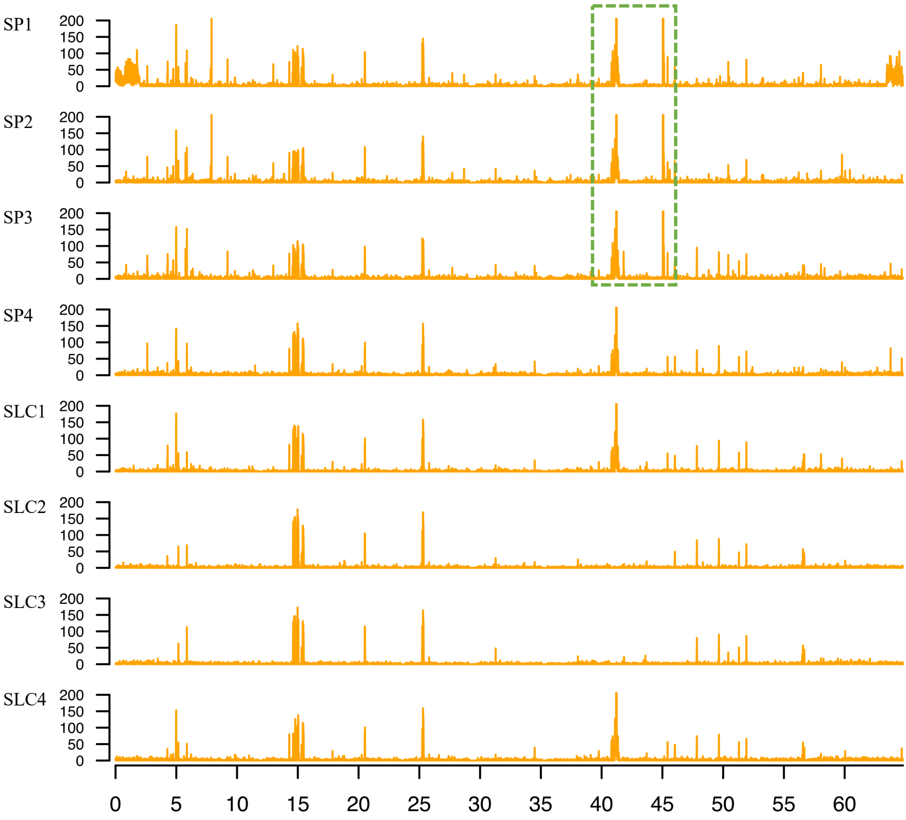

Chromosome 11 heterozygous variants

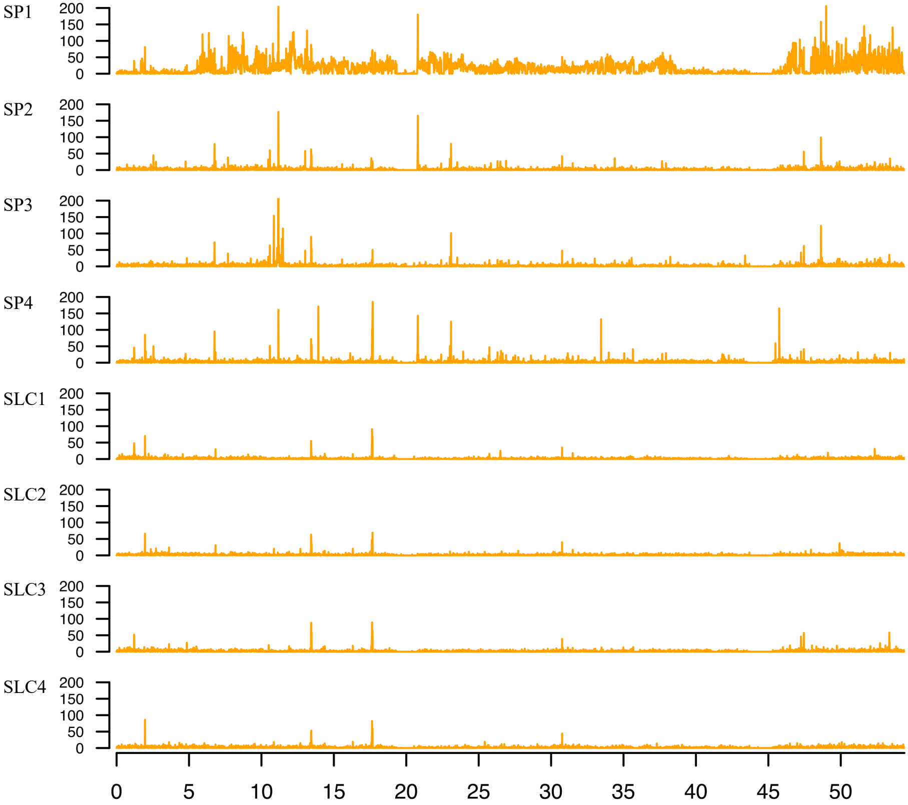

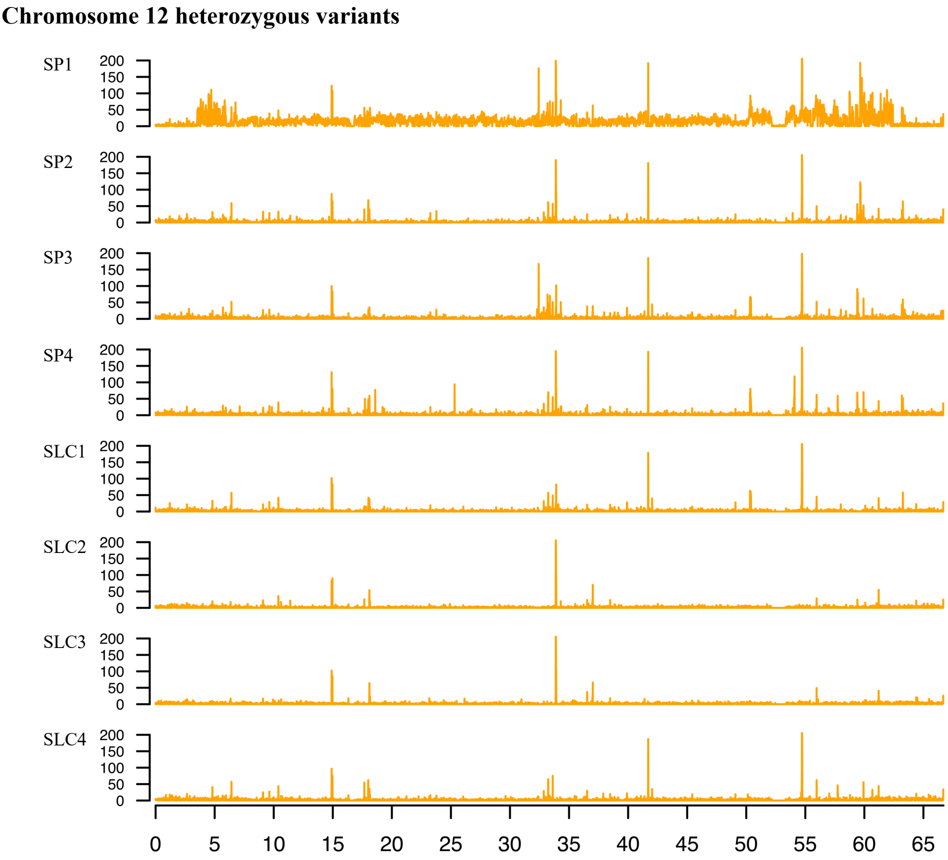

Chromosome 12 heterozygous variants
SP1
SP2
SP3
SP4
SLC1
SLC2
SLC3
SLC4
200
150
100
50
0
0
5
10
15
20
25
30
35
40
45
50
55
60
65
